# Information processing in the NF-κB pathway

**DOI:** 10.1038/s41598-017-16166-y

**Published:** 2017-11-21

**Authors:** Karolina Tudelska, Joanna Markiewicz, Marek Kochańczyk, Maciej Czerkies, Wiktor Prus, Zbigniew Korwek, Ali Abdi, Sławomir Błoński, Bogdan Kaźmierczak, Tomasz Lipniacki

**Affiliations:** 1 Institute of Fundamental Technological Research, Polish Academy of Sciences, Warsaw, Poland; 2 Department of Biological Sciences and Department of Electrical and Computer Engineering, New Jersey Institute of Technology, New Jersey, United States of America

## Abstract

The NF-κB pathway is known to transmit merely 1 bit of information about stimulus level. We combined experimentation with mathematical modeling to elucidate how information about TNF concentration is turned into a binary decision. Using Kolmogorov-Smirnov distance, we quantified the cell’s ability to discern 8 TNF concentrations at each step of the NF-κB pathway, to find that input discernibility decreases as signal propagates along the pathway. Discernibility of low TNF concentrations is restricted by noise at the TNF receptor level, whereas discernibility of high TNF concentrations it is restricted by saturation/depletion of downstream signaling components. Consequently, signal discernibility is highest between 0.03 and 1 ng/ml TNF. Simultaneous exposure to TNF or LPS and a translation inhibitor, cycloheximide, leads to prolonged NF-κB activation and a marked increase of transcript levels of NF-κB inhibitors, IκBα and A20. The impact of cycloheximide becomes apparent after the first peak of nuclear NF-κB translocation, meaning that the NF-κB network not only relays 1 bit of information to coordinate the all-or-nothing expression of early genes, but also over a longer time course integrates information about other stimuli. The NF-κB system should be thus perceived as a feedback-controlled decision-making module rather than a simple information transmission channel.

## Introduction

Cell signaling is performed by pathways and networks. The pathways, conceptualized as linear compositions of biochemical signal transduction elements, can be perceived as communication channels that transmit information from stimuli to respective outputs^1^. Information relayed by a pathway is degraded due to stochasticity inherent in biochemical reactions (intrinsic noise), whereas the variability in cellular states (extrinsic noise) reduces information available to an observer who has no knowledge about the parameters governing information transmission through the channel^2^. Cell signaling networks, conceptualized as systems of densely interconnected components that employ nonlinear functional elements such as feedbacks and delays, are perceived as functional modules capable of not only information transmission but also processing.

The aim of information processing is to convert incoming signals into one of predefined cellular responses. These responses can either be graded or have a form of digitized decisions (or physiological “programs”), such as proliferation, apoptosis, senescence, differentiation, epithelial–mesenchymal transition, autophagy, entosis, and others^3^. Whether to survive or commit to apoptosis is an example of a binary cell fate decision that is consequent upon the collection of a wide range of information^4^. Reaching such a binary decision results from integration and processing of information rather than its trivial degradation to ultimately 1 bit just by noise.

The capacity of an information channel is the number of bits that can be transmitted per unit of time. Shannon formally defined channel capacity as an upper bound on mutual information that can be transmitted over a sufficiently long time, *T*, divided by *T* ^5,6^. If input has the form of pulses, information capacity of the channel can be estimated as the number of bits transmitted in a single pulse multiplied by the maximal number of pulses per time unit. Werner *et al*.^7,8^ showed in mouse embryonic fibroblasts (MEFs) that NF-κB can translocate to the nucleus in response to TNF pulses as short as 1 min, and a consequent pulse of nuclear NF-κB peaks between 15 and 30 min and lasts for about 1 hour after TNF stimulation. Later, Ashall *et al*.^9^ showed in SK-N-AS cells that NF-κB exhibits translocation in response to 5-min pulses repeated every 200, 100, 60 min; however, only for the lowest frequency are the amplitudes of the first and the subsequent pulses equal. The information capacity of the NF-κB channel is thus determined by the refractory time, i.e., the minimal time after which the system is capable of responding to a subsequent pulse. Refractory time of the NF-κB channel appears to be cytokine-dependent^10^. Recently, using an experiment-calibrated model, we found that NF-κB has the ability to respond to sequences of TNF pulses of amplitude 10 ng/ml (“true” pulses) or 0 ng/ml (“false” pulses) with a frequency lower than 1/hr, exhibiting nuclear translocation after “true” pulses and ignoring “false” pulses^11^. This model prediction awaits experimental verification.

For the pulsatile TNF stimulation protocols, the NF-κB channel capacity is thus determined by the number of bits that can be transmitted in a single pulse and pulse frequency. As the first NF-κB pulse is very similar for both tonic and pulsed TNF stimulation, the value obtained by Cheong *et al*.^1^ for tonic stimulation can be considered as an appropriate estimate of the number of bits transmitted in a single pulse: they found that the NF-κB response can yield at most 0.92 ± 0.01 bit of information. This is equivalent to resolving 2^0.92^ ≈ 2 concentrations of the TNF signal, which is equivalent to reporting only whether TNF is present or not. Together with the predicted refractory time, this imposes an upper bound on the NF-κB channel information transmission at 1 bit/hr.

Here, we analyze how information transmitted through subsequent steps of the signaling cascade from TNF receptor (TNFR) to NF-κB is reduced or turned into a binary decision. We employ our recently calibrated^11^ mathematical model of the NF-κB network^12–14^ and focus on the first pulse of NF-κB translocation. In addition to estimating upper bound of mutual information, we quantify the ability of the system to discern the neighboring concentrations of a stimulus at each step of the core pathway by means of the Kolmogorov–Smirnov distance, a measure of distance between two probability distributions^15^.

## Results

### Interpretation of confocal image data and computational model assumptions

**Figure 1:**
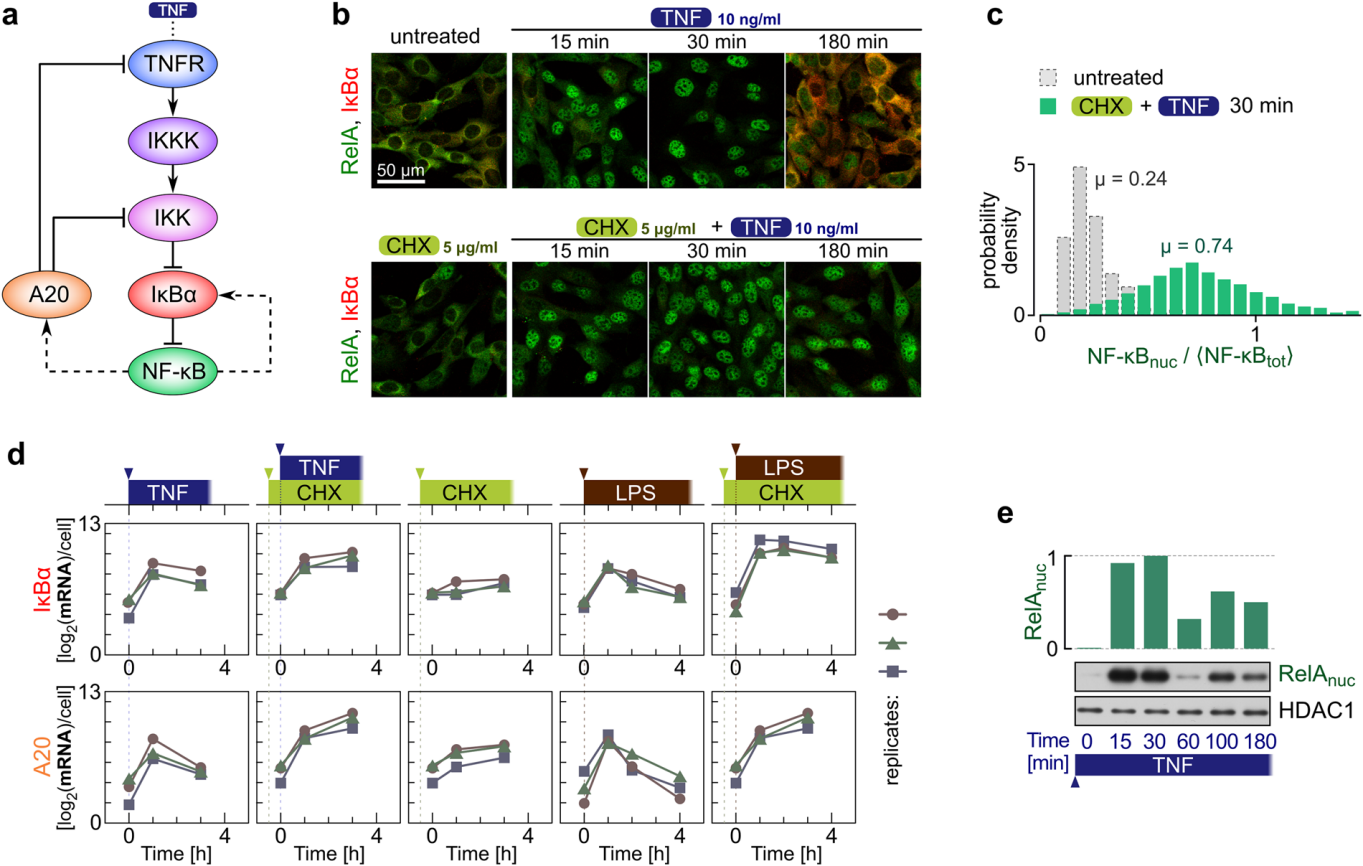
Experimental characterization of NF-κB response to TNF and LPS under conditions of normal or inhibited translation. MEFs were stimulated with 10 ng/ml TNF or 1 μg/ml LPS in the absence or presence of 5 μg/ml cycloheximide (CHX). In the CHX + TNF and CHX + LPS costimulation experiments, cells were incubated with 5 μg/ml CHX for 30 min prior to TNF or LPS stimulation. (**a**) Schematic diagram of the NF-κB pathway. A detailed diagram can be found in Korwek *et al*.^11^. (**b**) Temporal coevolution of nuclear NF-κB and total IκBα. Following stimulation, cells were fixed and stained with antibodies for RelA (a subunit of NF-κB) and for IκBα. Representative excerpts from confocal images show cells at 0 (‘untreated’), 15, 30 and 180 min after TNF stimulation. See Supplementary Data S1 for corresponding uncropped immunostaining images. (**c**) Histograms (*n* = 466 for untreated cells and *n* = 1434 for CHX + TNF costimulated cells) show NF-κB nuclear translocation, defined as nuclear fluorescence normalized to the average whole-cell fluorescence, based on confocal images (see Methods for details). The CHX + TNF histogram is derived from confocal images for the 30-min time point of the CHX + TNF costimulation experiment, which corresponds to the observed peak of NF-κB nuclear translocation. µ denotes the mean values of the distributions. (**d**) Gene expression profiles of NF-κB inhibitors, IκBα and A20. Time profiles of relative mRNA levels of IκBα and A20 were obtained using digital PCR measurements. (**e**) Western blot of nuclear RelA (a subunit of NF-κB) in response to TNF and its densitometric quantification with HDAC1 as reference. Average ratio of NF-κB_nuc_(*t* = 0)/NF-κB_nuc_(*t* = 30 min) calculated based on 3 such independent experiments is 0.03, which allows to estimate the nuclear NF-κB fraction at *t = *0, NF-κB_nuc_(*t* = 0)/NF-κB_total_, to be below 0.03. See Supplementary Data S2 for full-length Western blots.

Extrinsic noise, which influences information transmission in signaling networks, is associated with heterogeneity in concentrations of network components and variability of cellular states across a population of cells. Here, we examine the NF-κB network (a simplified network scheme is shown in Fig. 1a) and, following our earlier study^13^, we take into account only the variability in the levels of TNFR and NF-κB. As the TNFR distribution is unknown, it is considered as a free parameter of the model. Finding the distribution of NF-κB in a cell population based on confocal images is difficult since cell boundaries are not clearly visible. In the context of modeling, an important variable is however not the total but the “translocatable” NF-κB, i.e., the NF-κB that can translocate to the nucleus after being released from IκB isoforms degraded in response to TNF. In our model, IκBα stands for all IκB isoforms. Experimental data suggest that even after almost all IκBα is degraded, a noticeable fraction of NF-κB remains cytoplasmic (Fig. 1b), possibly because it is sequestered by other inhibitors that are not degraded in response to TNF or because some post-translational modifications preclude its nuclear translocation.

We estimate the distribution of translocatable NF-κB in the cell population by determining the distribution of nuclear NF-κB after 30 min of 10 ng/ml TNF stimulation in cells pretreated with cycloheximide (CHX; Fig. 1c, green bars). By inhibiting translation, CHX blocks the synthesis of NF-κB inhibitors, IκBα and A20. In CHX-pretreated cells, IκBα (*Nfkbia*) and A20 (*Tnfaip3*) mRNA accumulate over 3–4 hours after TNF or LPS stimulation (Fig. 1d), indicating transcriptional activity, however, no IκBα proteins are visible in immunostaining images at the end of that period, and most of NF-κB reside in the nucleus (Fig. 1b), as also observed by Sung *et al*.^16^. Notwithstanding, in cells stimulated with TNF or LPS without CHX pretreatment, IκBα mRNA profile peaks at about 1 hour, and IκBα protein accumulates over 3 hours and directs NF-κB out of the nucleus. Thus, we expect that in conditions of inhibited translation virtually all translocatable NF-κB is localized to the nucleus.

Quantification of immunofluorescence data indicates that the average fraction of nuclear NF-κB in untreated cells is 0.24 (Fig. 1c, gray bars). However, according to Western blotting data, the level of nuclear NF-κB in untreated cells is about 0.03 of the maximal nuclear NF-κB in TNF-stimulated cells, which implies that the nuclear fraction in unstimulated cells is below 0.03 (Fig. 1e). We propose that this discrepancy between microscopy and blotting data arises from the fact that about 0.24 of cytoplasmic immunofluorescence, resulting from the presence of NF-κB below and above the nucleus, registers as nuclear. We further refer to this microscopy technique error as the cytoplasmic interference (CI). In order to juxtapose numerical simulations and experimental data, the former are modified to account for CI, whereas experimental data are presented without any modification. The magnitude of CI is very likely cell-dependent, however measuring it in single cells would be very difficult as it would require estimation of nuclear NF-κB (at the single-cell level) by a method different than immunostaining.

Having applied the CI correction to the average nuclear NF-κB fraction observed in response to TNF and CHX costimulation and equal *x*_obs_ = 0.74 (Fig. 1c), we deduce the actual nuclear NF-κB fraction *x*_max_ using the formula *x*_obs_ = *x*_max_ + 0.24(1 − *x*_max_), which gives *x*_max_ = (0.74 − 0.24)/(1 − 0.24) ≈ 2/3. Based on it, we estimate the ratio of the translocatable NF-κB pool to the inert NF-κB pool as 2:1, and assume that both NF-κB types are distributed independently according to the experimental NF-κB distribution obtained for CHX and TNF costimulation. In the computational model we assume that on average there are 10^5^ NF-κB molecules in the translocatable pool and 0.5 × 10^5^ NF-κB molecules in the inert pool. The latter is only used to normalize the amount of nuclear NF-κB in each cell by dividing it by an average cellular NF-κB in the population of cells (see Methods). Such normalization is needed to reproduce experimental results showing that no more than 2/3 of NF-κB translocate to the nucleus.

### Mutual information

Mutual information, MI, of two discrete random variables X and Y can be defined as

1 $${\rm{MI}}(X;Y)=\sum _{y\in Y}\sum _{x\in X}p(x,y)\mathrm{log}(\frac{p(x,y)}{p(x)p(y)}),$$

where *p*(*x*,*y*) is the joint distribution of outputs *X* and inputs *Y*. Inputs *Y* will be identified with 8 TNF stimulation doses, while outputs *X* with the levels of selected components of the NF-κB pathway, at a given time point. Throughout the article, the default output is the level of nuclear NF-κB; in the subsection “Mutual information and dose discernibility at each level of the NF-κB pathway” we consider additionally five other pathway components shown in the scheme in Fig. 1a. Formally, outputs are discrete since we quantify each protein level as the number of molecules per cell. MI is estimated according to the method proposed by Kraskov *et al*.^17^ (see Supplementary Dataset S2 for a python implementation). The upper bound for transmitted information is estimated by maximizing MI with respect to 8 input (or “*a priori*”) probabilities *p*(*y*) using a steepest-ascent method. More specifically, Eq. (1) can be re-written as

2 $${\rm{MI}}(X;Y)=\sum _{y\in Y}p(y)\sum _{x\in X}p(x|y)\mathrm{log}(\frac{p(x|y)}{p(x)}),$$

where $p(x|y)=p(x,y)/p(y)$ represents the distribution of the output *x* conditioned on the input *y*. Let ${p}_{i}=p(y={{\rm{TNF}}}_{i})$ be the probability for input dose TNF_*i*_, *i* = 1, …, 8. This simplifies Eq. (2) to

3 $${\rm{MI}}(X;Y)=\sum _{i=1}^{8}{p}_{i}\sum _{x\in X}p(x|{{\rm{TNF}}}_{i})\mathrm{log}(\frac{p(x|{{\rm{TNF}}}_{i})}{p(x)}),$$

where ${\sum }_{i=1}^{8}{p}_{i}=1$. When we consider pairs of TNF input concentrations TNF_1_ and TNF_2_ with probabilities *p*_1_ + *p*_2_ = 1 (Supplementary Data S4), the index of the outer sum runs over *i* = 1, 2.

We should notice that since our analysis is based on sampled one-dimensional probability distributions, we quantify how much information on average is transmitted by a single cell in which NF-κB response is measured at a single time point. Similarly using Kolmogorov–Smirnov statistics (introduced below) we will quantify “average single cell” ability to discern two stimulation doses.

### Dose discernibility

**Figure 2:**
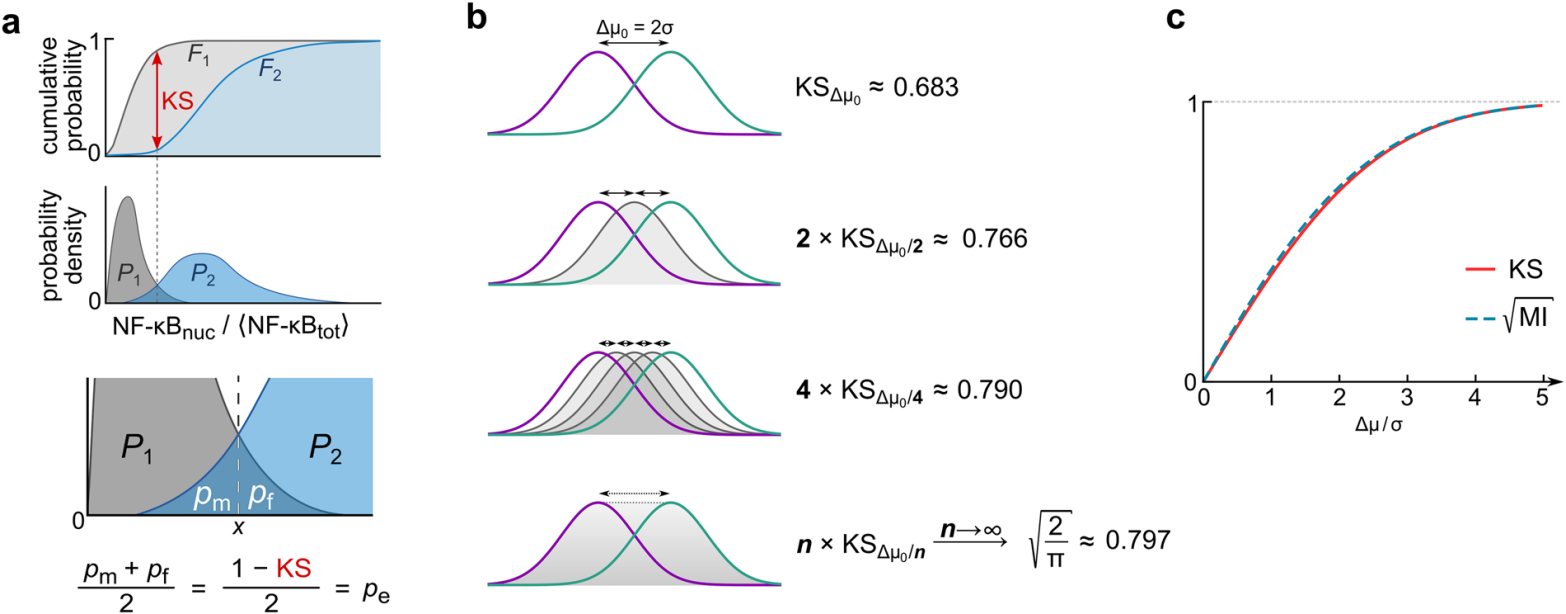
Concept of dose discernibility. (**a**) Correspondence between the Kolmogorov–Smirnov distance (KS) and the miss and false alarm probabilities. Absolute difference between cumulative distribution functions |*F*_1_(*x*) − *F*_2_(*x*)| reaches its supremum, called KS distance, in point *x* for which probability densities of distributions *P*_1_ and *P*_2_ are equal, *P*_1_(*x*) = *P*_2_(*x*). When the prior probabilities corresponding to these distributions are equal, then point *x* is the optimal decision threshold, defining miss and false alarm probabilities, *p*_m_, *p*_f_. (**b**) Sum of KS distances between Gaussian distributions in between two Gaussians with Δµ_0_ = 2σ. (**c**) Comparison of $\sqrt{{\rm{MI}}}$ and KS, see text for details.

To quantify the dose discernibility at different levels of the pathway we will use the Kolmogorov–Smirnov distance (KS). KS can be used to quantify the distance between two empirical distribution functions, *P*_1_ and *P*_2_, of two samples, *S*_1_ and *S*_2_, as

4 $$\mathrm{KS}({P}_{1},{P}_{2})=\mathop{\sup }\limits_{x}|{F}_{1}(x)-{F}_{2}(x)|,$$

where *F*_1_ and *F*_2_ are cumulative distributions of *P*_1_ and *P*_2_ and sup is the supremum of the set of distances (Fig. 2a). In our cases, samples are cell populations stimulated with different TNF concentrations. In the cases analogous to this shown in Fig. 2a, KS distance between *P*_1_ and *P*_2_ has a simple graphical representation: 1 − KS is the shared area below both probability density functions. KS distance has two properties that make it a useful metric to quantify how well a given pair of doses can be discerned by measuring corresponding outputs: (*i*) KS can be interpreted in terms of miss and false alarm probabilities, (*ii*) KS satisfies the triangle inequality which permits its use as a metric for measuring discernibility of consecutive stimulation doses.

(***i***) Let us notice that the shared area below the distribution functions *P*_1_ and *P*_2_ can be divided into two subareas by the line perpendicular to the *x*-axis, passing through point *x* such that *P*_1_(*x*) = *P*_2_(*x*) (see Fig. 2a). When the prior probabilities of two doses are equal, *x* is an optimal decision threshold^18^. When both samples are sufficiently large (precisely, infinitely large), these two subareas correspond to the *miss* and *false alarm* probabilities, *p*_m_ and *p*_f_. In our case, miss probability is the probability that a cell from sample *S*_2_ is misinterpreted as a cell originating from *S*_1_, and, conversely, the false alarm probability is the probability that a cell from sample *S*_1_ is misinterpreted as a cell originating from *S*_2_. When both miss and false alarm probabilities are 0, then KS = 1 − *p*_m_ − *p*_f_ = 1, and one can deduce with certainty which one of two stimulation concentrations was applied to an observed cell. Generally, when prior probabilities are equal, the error probability, i.e., probability of inappropriate dose assignment for a randomly selected cell, is

5 $${p}_{{\rm{e}}}=\frac{{p}_{{\rm{m}}}+{p}_{{\rm{f}}}}{2}=\frac{1-{\rm{KS}}}{2}.$$

Of note, *p*_m_ and *p*_f_ were recently used to quantify decision making errors in the NF-κB system^18^.

(***ii***) KS satisfies the triangle inequality, i.e., KS(*a*, *c*) ≤ KS(*a*, *b*) + KS(*b*, *c*). Let us consider two Gaussian distributions: *N*_1_ *= N*(*x*; µ_1_, σ^2^) and *N*_2_ *= N*(*x*; µ_2_, σ^2^) with the same variance σ^2^ and means satisfying |µ_1_ − µ_2_| = Δµ. To analyze how KS can be used to discern close distributions we calculate $\mathop{\mathrm{lim}}\limits_{\Delta {\rm{\mu }}\to {\rm{0}}}\mathrm{KS}({N}_{{\rm{1}}},{N}_{2})\frac{{\rm{\sigma }}}{{\rm{\Delta }}{\rm{\mu }}}$. Without losing generality we may assume that µ_1_ = Δµ/2 and µ_2_ = −Δµ/2. Now,

6 $$\mathop{\mathrm{lim}}\limits_{\Delta {\rm{\mu }}\to {\rm{0}}}\mathrm{KS}({N}_{{\rm{1}}},{N}_{2})\frac{{\rm{\sigma }}}{{\rm{\Delta }}{\rm{\mu }}}=\frac{1}{2}\mathop{\mathrm{lim}}\limits_{\Delta {\rm{\mu }}\to {\rm{0}}}\frac{{\rm{\sigma }}}{{\rm{\Delta }}{\rm{\mu }}}[{\rm{erf}}(\frac{{\rm{\Delta }}{\rm{\mu }}}{{\rm{2}}\sqrt{{\rm{2}}}{\rm{\sigma }}})-{\rm{erf}}(\frac{-{\rm{\Delta }}{\rm{\mu }}}{{\rm{2}}\sqrt{{\rm{2}}}{\rm{\sigma }}})]=\frac{{\rm{\sigma }}}{{\rm{\Delta }}{\rm{\mu }}}{{\rm{\Delta }}{\rm{\mu }}N(0;0,{\rm{\sigma }}}^{{\rm{2}}})=\frac{{\rm{1}}}{\sqrt{{\rm{2}}\pi }}\cong 0.398,$$

where we make use of the facts, that the maximal distance between the cumulative distributions (KS) is attained in the point in which corresponding distributions are equal (in this case at *x* = 0) and that the derivative of erf function at *x* = 0 is equal to 2 × *N*(0; 0, σ^2^). Correspondingly, the sum of KS distances between distributions with equidistant means located in-between two limiting distributions separated by Δµ_0_ (as shown in Fig. 2b) converges to $(1/\sqrt{{\rm{2}}\pi }){{\rm{\Delta }}{\rm{\mu }}}_{{\rm{0}}}/{\rm{\sigma }}$ as the number of intermediary distributions tends to infinity. Thus, incremental KS distances can be used to measure length of a “path” between two distributions in an analogous way as segments can be used to measure the length of a curve between two points. Based on the above example, one can define adjacent inputs discernibility *C*(*d*) as

7 $$C(d)=\mathop{\mathrm{lim}}\limits_{{d}_{1}\to d}\frac{{\rm{KS}}({P}_{1},P)}{{d}_{1}-d},$$

where *P* and *P*_1_ are probability functions corresponding to the consecutive doses *d* and *d*_1_. The doses can be in the logarithmic scale. The example from Fig. 2b also suggests that, since convergence in Eq. (6) is fast, the local dose discernibility, Eq. (7), is well approximated by the difference quotient

8 $$C(d)\cong \frac{{\rm{KS}}({P}_{1},P)}{{d}_{1}-d}$$

when roughly KS(*P*_1_, *P*) < 0.4. Local system sensitivity can be defined as

9 $$S(d)=\mathop{\mathrm{lim}}\limits_{{d}_{1}\to d}\frac{\mathrm{mean}({P}_{{\rm{1}}})-\mathrm{mean}(P)}{{d}_{1}-d}.$$

Eq. (9) allows to interpret *C*(*d*) as a measure proportional to *S*(*d*)/σ(*d*) and implies that for Gaussian distributions the proportionality coefficient is ${\rm{1}}/\sqrt{{\rm{2}}\pi }$.

Finally, let us notice that $\sqrt{{\rm{MI}}({\rm{\Delta }}{\rm{\mu }}/{\rm{\sigma }})}\approx {\rm{KS}}({\rm{\Delta }}{\rm{\mu }}/{\rm{\sigma }})$, where ${\rm{MI}}(\Delta {\rm{\mu }}/{\rm{\sigma }})$ denotes the mutual information between two inputs of probabilities *p*_1_ = *p* = 1/2 and corresponding Gaussian outputs, *N*_1_(µ_1_, σ^2^) and *N*(µ, σ^2^), with the same variance σ^2^ and means satisfying |µ_1_ – µ| = Δµ, while ${\rm{KS}}({\rm{\Delta }}{\rm{\mu }}/{\rm{\sigma }}):=\mathrm{KS}({N}_{1},N)$, see Fig. 2c. Let us also notice that when outputs are Gaussians with same variance σ^2^, then the maximal value of MI is reached when *p*_1_ = *p* = 1/2, which is however not true for distributions of different variances. Although this approximate relation is not universal, proportionality between maximized $\sqrt{{\rm{MI}}}$ and KS is surprisingly well held (with the Pearson correlation coefficient greater than 0.99) for both experimental and model-simulated distributions (see Supplementary Data S4). Because for close doses MI ≪ KS, the latter can be calculated with a smaller relative error.

### Analysis of nuclear NF-κB response to eight TNF concentrations

**Figure 3:**
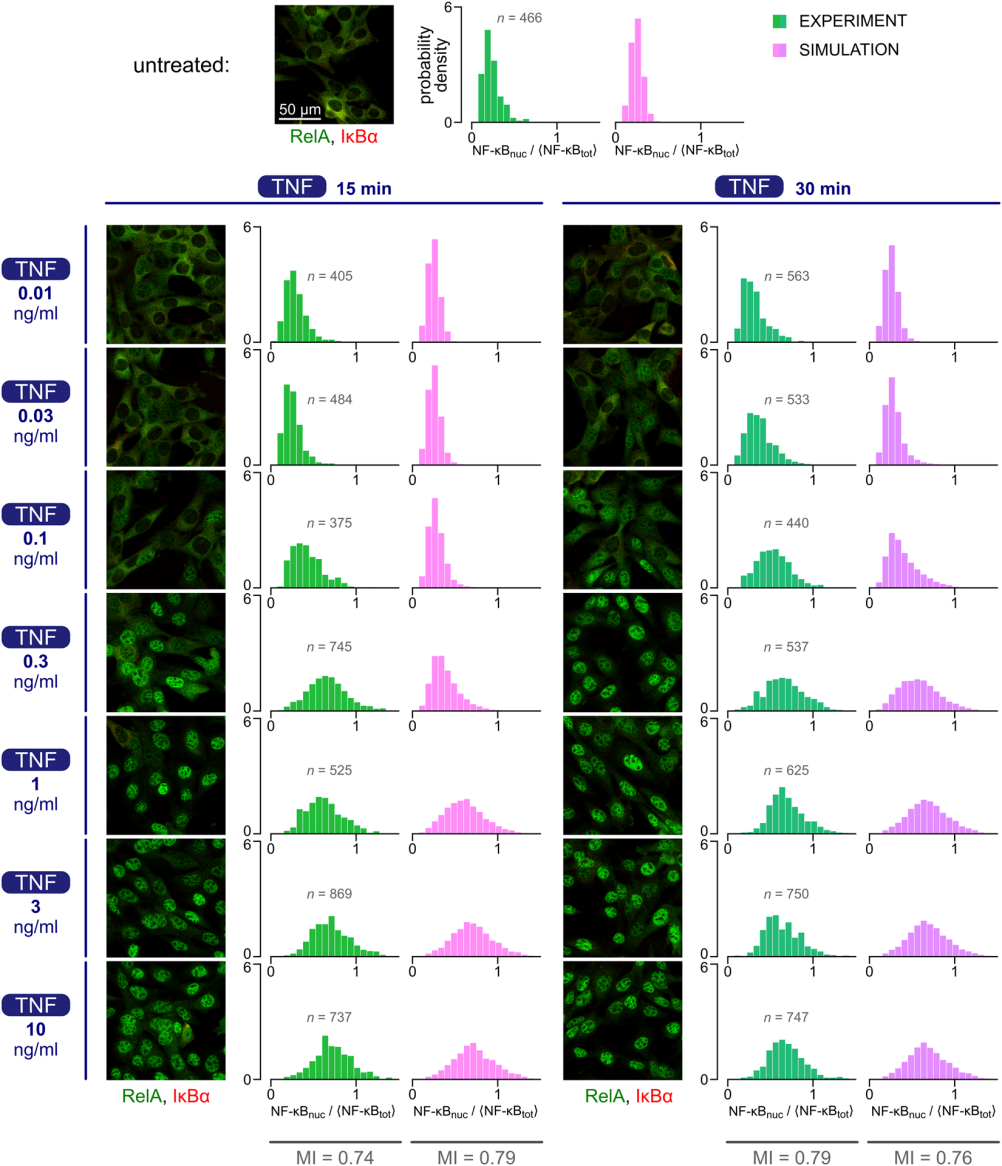
Nuclear NF-κB response to eight TNF concentrations: experiment and model. MEFs were stimulated with 0, 0.01, 0.03, 0.1, 0.3, 1, 3 or 10 ng/ml TNF, fixed and stained with antibodies for RelA (a subunit of NF-κB) and IκBα. Representative regions from confocal images show cells at 15 min (left) and 30 min (right) of TNF stimulation. See Supplementary Data S3 for corresponding uncropped immunostaining images. Histograms show NF-κB nuclear translocation calculated based on confocal images (green) and on numerical simulations (pink) for each combination of TNF stimulation time and concentration (the number of cells, *n*, quantified in each experiment, is shown next to each histogram, the number of stochastic simulations for each TNF dose is 10,000). NF-κB nuclear translocation is defined as the nuclear fluorescence normalized to the average whole-cell fluorescence (see Methods for quantification details). Simulations take into account cytoplasmic interference (CI) in order to enable comparison with experimental results.

NF-κB translocation in response to eight TNF concentrations (0, 0.01, 0.03, 0.1, 0.3, 1, 3 and 10 ng/ml) was measured using immunofluorescence 15 min and 30 min after stimulation and juxtaposed with model simulations (Fig. 3). The experimental histograms of normalized nuclear NF-κB show that the maximal change of the distributions occurs between 0.03 and 0.3 ng/ml TNF. Nuclear translocation of NF-κB is lower at 15 min than at 30 min for TNF concentrations below 1 ng/ml. For both time points, saturation is reached at 1 ng/ml TNF, and the responses at these two time points become nearly indistinguishable. Weaker responses at 15 min are in agreement with the delayed NF-κB activation in response to low-concentration stimulation, as reported previously for both TNF and LPS^19^.

When comparing model simulations with experiment, we account for the cytoplasmic interference. Qualitatively, experimental and simulation-based histograms are similar, but the latter show the maximal change of the distributions for somewhat higher TNF doses. Correspondingly, the simulated and experimental KS distances between all TNF dose pairs follow the same pattern with higher experimental KS values for low TNF stimulation (Supplementary Data S4). The MI values at the level of NF-κB, maximized with respect to eight input probabilities, are very close for the experiment and the model: 0.74 versus 0.79 for 15 min; 0.79 versus 0.76 for 30 min (Fig. 3). For both the model and the experiment, all KS distances are smaller than 1, which means that even unstimulated cells and cells stimulated with the highest TNF dose (10 ng/ml) may not be distinguished with absolute certainty. In this case, KS distance reaches about 0.9 (for both the experiment and the model, see Supplementary Data S4) which is consistent with the miss and false alarm probabilities reported by Habibi *et al*.^18^, who estimated *p*_m_ = 0.1 and *p*_f_ = 0.04, which implies KS = 0.86.

In the following sections, based on the computational model, we analyze:

- how the dose discernibility and MI are obscured by cytoplasmic interference,
- how the dose discernibility and MI depend on the magnitude of extrinsic noise,
- how information is reduced when passing through consecutive steps of the signal transduction pathway.

### Interference from cytoplasmic fluorescence obscures transmitted information

**Figure 4:**
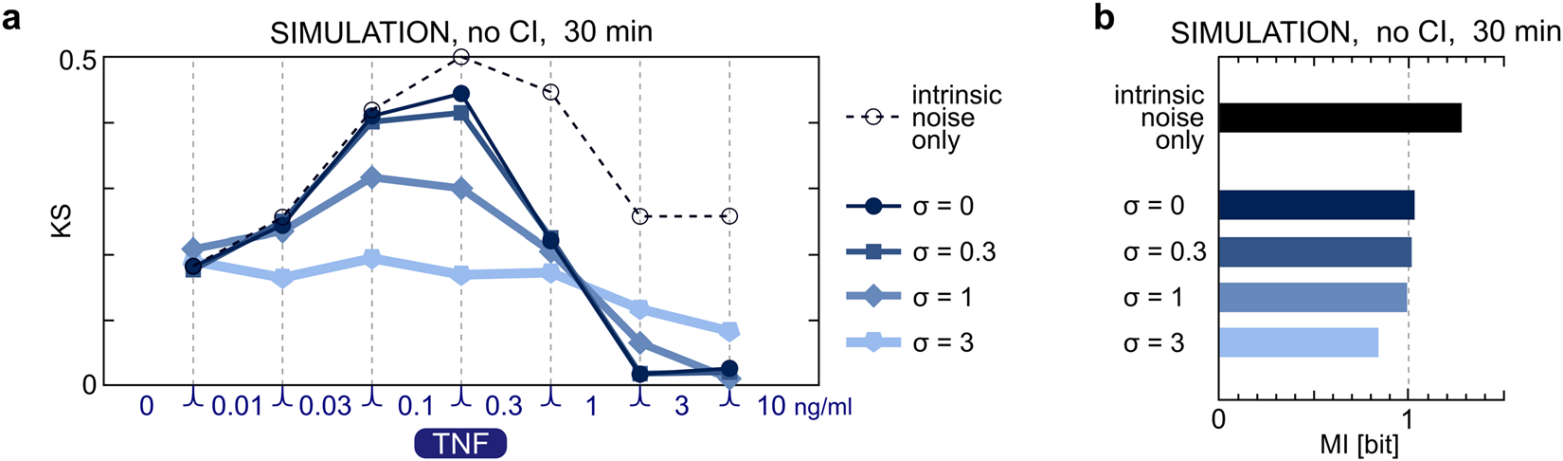
TNF dose discernibility and mutual information with respect to the extrinsic noise. (**a**) Plots show Kolmogorov–Smirnov (KS) distances between simulated distributions of nuclear NF-κB in 30-min time point of stimulation with consecutive TNF concentrations. The results are shown for standard deviation of TNFR, σ, equal 0, 0.3, 1 or 3. ‘Intrinsic noise only’ denotes simulations in which σ = 0, and additionally the same amount of total translocatable and nontraslocatable NF-κB is assumed for each cell. KS distances were calculated based on simulations without cytoplasmic interference (CI). CI reduces KS distances as shown in Supplementary Data S5. (**b**) The effect of extrinsic noise on mutual information at the NF-κB level in 30-min time point of TNF stimulation.

Cytoplasmic interference introduces an inherent bias in measurements of nuclear fluorescence, increasing its value. Thus, to compare model predictions with experiment, we accounted for cytoplasmic interference as described in Methods. Here, based on the model, we analyze how much information is lost from the observer’s perspective due to this bias of the experimental technique. The overall MI calculated for the model without cytoplasmic interference is about 1.0 bit (for σ = 0, 0.3, 1; see Fig. 4b) which is higher than for the model with cytoplasmic interference and higher than the value for experimental measurements, about 0.8 bit (Fig. 3). Also the KS distances between neighboring TNF concentrations without cytoplasmic interference are higher than with cytoplasmic interference (Supplementary Data S5). This difference is markedly pronounced at low stimulation concentrations, for which the level of nuclear NF-κB is low, and cytoplasmic interference contributes largely to nuclear fluorescence. As a result, small changes of nuclear NF-κB levels between small stimulus concentrations cannot be detected, which markedly reduces KS values between 0 and 0.01 ng/ml TNF. For higher concentrations, the effect of cytoplasmic interference is much less pronounced. This analysis suggests that the amount of information available from immunofluorescent images underestimates the actual amount of information available to cells. In further considerations we will thus analyze model simulations in which cytoplasmic interference is not taken into account.

### Transmission of information and dose discernibility are reduced by extrinsic noise

We consider two sources of extrinsic noise in the model, one associated with heterogeneity in the level of TNFR and the other associated with heterogeneity in the translocatable and nontranslocatable pools of NF-κB. We assume that TNFR is distributed according to the log-normal distribution *LnN*(µ, σ^2^) with constant µ (equivalently, constant median equal e^µ^) and varied σ, i.e., varied variance,${\rm{Var}}=({e}^{{{\rm{\sigma }}}^{2}}-1){e}^{{{\rm{\sigma }}}^{2}}\times {e}^{2{\rm{\mu }}}$. The distribution of translocatable and nontranslocatable NF-κB is estimated based on Fig. 1c, as described before. In Fig. 4a we provide KS distances between consecutive TNF concentrations, and overall MI for four values of σ: 0, 0.3, 1, 3, as well as in the hypothetical case when there is no extrinsic noise, i.e., when σ = 0 and the pools of translocatable and nontranslocatable NF-κB are invariant in all simulations (10^5^ and 5 × 10^4^, respectively). We call this case ‘hypothetical’ as we know from experimental data that the level of NF-κB varies across the cell population. Almost all KS distances decrease with σ. Interestingly, distances between highest concentrations are increased for σ = 3. This is because for σ = 3 some cells have a very low abundance of TNFR, so they respond differently to TNF concentrations of 1, 3, and 10 ng/ml, while for smaller σ almost all cells reach the saturation of the response. Although the increased width of the TNFR distribution introduces some uncertainty about the signal, it widens the dynamical range of the NF-κB pathway. This may explain relatively low sensitivity of MI to σ; MI remains close to 1 bit for σ = 0, 0.3, 1 and still exceeds 0.8 bit for σ = 3 (Fig. 4b). One cannot thus determine the width of TNFR distribution by measuring MI at the NF-κB level. Unsurprisingly, the highest MI, nearly 1.3 bit, is reached when no extrinsic noise is present.

We demonstrated that extrinsic noise may significantly reduce dose discernibility. The number of activated TNF receptors in a given cell is roughly proportional to the product of TNF concentration and the total number of TNFR on the cell membrane. From the perspective of an experimentalist who observes a single responding cell, it cannot be recognized whether it is the TNF concentration that is high or whether it is the number of receptors on that cell that is particularly large so the cell can respond strongly even to a low concentration. Similarly, the observer focused solely on the cell nucleus cannot tell whether nuclear NF-κB level is high because of the strong TNF signal or because the total NF-κB level is higher than its average in cell population. In the case of small extrinsic noise, σ ≤ 1, the highest adjacent doses discernibility *C*(*d*) is reached between 0.1 and 0.3 ng/ml TNF. In the case with no extrinsic noise, KS value between 0.1 and 0.3 ng/ml TNF is about 0.50, which allows to estimate maximal *C*(*d*) ≥ 0.5/log(3) ≈ 0.455; in the presence of extrinsic noise smaller values of *C*(*d*) are obtained.

### Mutual information and dose discernibility at each level of the NF-κB pathway

**Figure 5:**
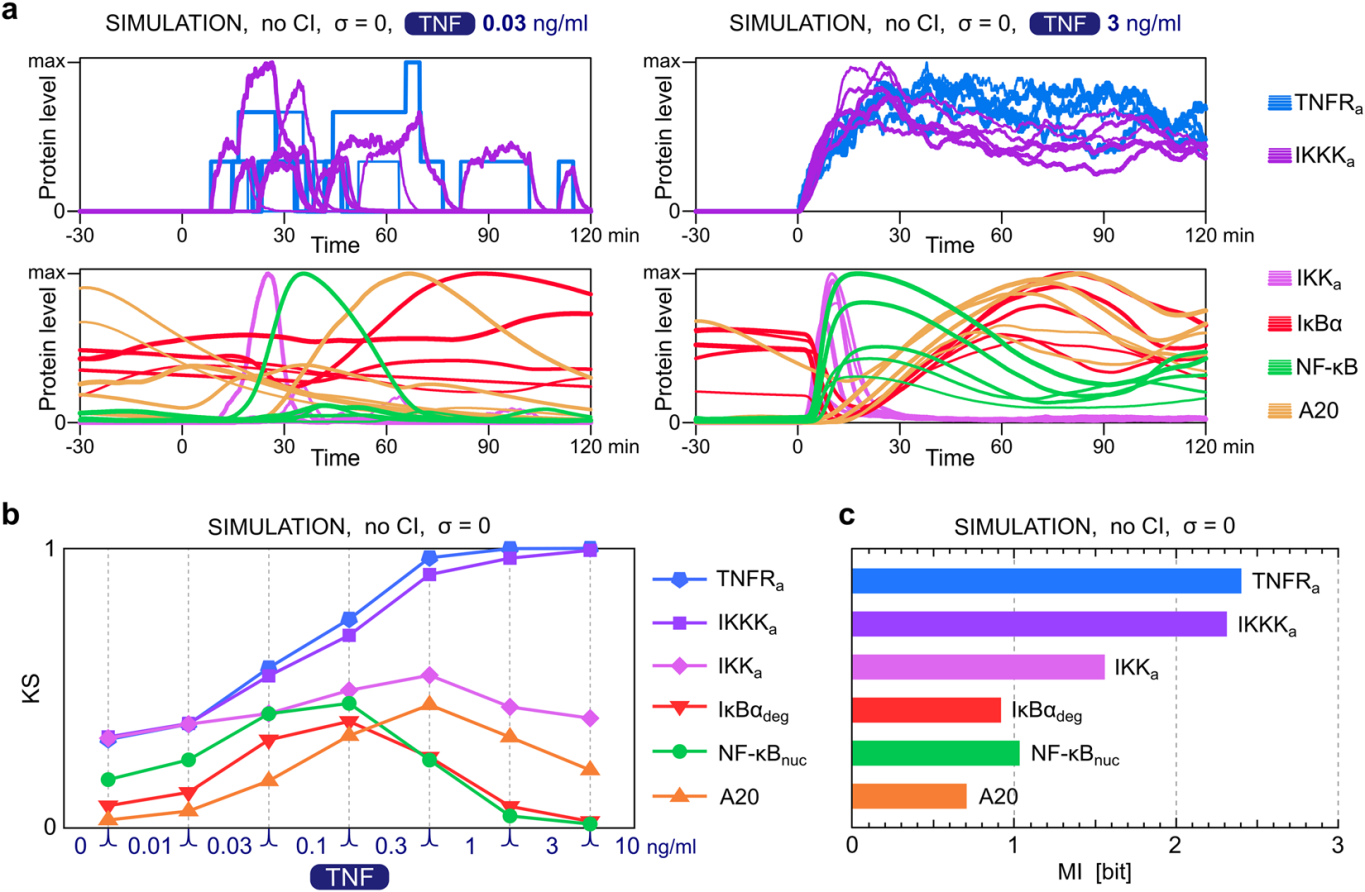
Information at each level of the NF-κB pathway. (**a**) Temporal profiles of key pathway components obtained in numerical simulations with σ = 0 for two TNF doses: 0.03 ng/ml and 3 ng/ml. In each subpanel, shown are normalized trajectories from 5 stochastic simulations (with the assumed translocatable pools of NF-κB equal {0.5, 0.7, 0.9, 1.3, 1.6} × 10^5^ – probing the experimental distribution from Fig. 1c with the average of 10^5^ molecules). Trajectories with larger pools of translocatable NF-κB are drawn with thicker lines. (**b**) Kolmogorov–Smirnov (KS) distances between simulated distributions of pathway components calculated for each pair of consecutive TNF concentrations. (**c**) Mutual information at each pathway level. The results shown in (**b**) and (**c**) are based on maximal levels of the pathway components over 0–30 min after TNF stimulation, for *n* = 10,000 simulations with σ = 0, without CI. IκBα_deg_ denotes the maximal value of IκBα(*t = *0) − IκBα(*t*) for *t* $\in$ [0,30 min]. The analogous results for σ = 0.3, σ = 1 and σ = 3 and in the case with the intrinsic noise only, are shown in Supplementary Data S7.

In Fig. 5 we analyze how dose discernibility (Fig. 5b) and mutual information (Fig. 5c) are reduced when passing through the NF-κB pathway (Fig. 1a). Since the signal is relayed through subsequent steps of the pathway at different times, we calculated KS distances based on the peak values of active TNFR (TNFR_a_), active IKKK (IKKK_a_), active IKK (IKK_a_), degraded IκBα (IκBα_deg_), NF-κB_nuc_, and A20 – all within first 30 min after TNF stimulation. Example simulated trajectories of these components are provided for two TNF doses, 0.03 and 3 ng/ml in Fig. 5a. For the low dose, cell responses are highly heterogeneous due to molecular noise at the TNFR_a_ level. KS distances between 1 and 3, and 3 and 10 ng/ml, at the TNFR_a_ and IKKK_a_ levels are close to 1 (Fig. 5b), suggesting that information is accurately transmitted through the first two steps of the pathway for large TNF concentrations. However, for smaller TNF concentrations, KS distances are much lower, suggesting information loss. The overall MI at the level of TNFR_a_ or IKKK_a_ reaches about 2.3 bit (Fig. 5c), which may be compared to 3 bits, or 8 stimulation levels at input. Molecular noise (visible in trajectories shown in Fig. 5a for TNF dose of 0.03 ng/ml) is responsible for the system’s inability to accurately discern low TNF concentrations. As long as TNFR_a_ ≪ TNFR, the distribution of TNFR_a_ is close to Poisson distribution with its mean proportional to TNF concentration. Because the coefficient of variation of Poisson distribution is inversely proportional to the square root of the mean, TNFR_a_ distributions corresponding to low concentrations overlap (Supplementary Data S6) and corresponding KS distances are much smaller than 1 (Fig. 5b).

At the level of IKK_a_, the response saturation at high TNF concentrations becomes important, and KS values are substantially decreased with respect to these for IKKK_a_, which is not the case for low TNF concentrations (Fig. 5b). Compared to IKK_a_, a further substantial decrease in mutual information occurs at the level of IκBα_deg_ (Fig. 5c). A bit surprisingly, MI (as well as KS distances) measured at the NF-κB_nuc_ level are somewhat higher than at the level of IκBα_deg_. One should keep in mind that MI measured at the single time point in which IκBα_deg_ reaches its maximum does not quantify total transmitted information; downstream responses are influenced by the whole time profile of IκBα. Additionally, NF-κB_nuc_ depends rather on the degraded IκBα that was previously complexed with NF-κB, and not on the whole pool of degraded IκBα, which includes free IκBα and varies across cell population. Also, at high TNF concentrations, KS values at the level of A20 are higher than these for NF-κB_nuc_, because at high TNF concentrations NF-κB activation starts faster granting A20 more time to accumulate. The overall MI is however lower for A20 than for NF-κB_nuc_.

In Supplementary Data S7 we repeat the analysis shown in Fig. 5b,c for different values of σ, and for the case without extrinsic noise. Increasing σ reduces MI and dose discernibility mainly at the upper levels of the pathway; the effect is especially pronounced for TNFR_a_ and IKKK_a_ for which the MI is gradually reduced from about 2.3 bit to 0.9 bit as σ changes from 0 to 3. As indicated by the KS values between adjacent TNF doses, heterogeneity of total TNFR level dramatically reduces discernibility of high doses at the level of TNFR_a_ and IKKK_a_, which explains why information is reduced at the very beginning of the pathway. For σ = 0, the difference between the case in which distribution or single value of translocatable NF-κB is assumed is observed mainly at the at NF-κB level, and (as expected) is not observed above IκBα in the pathway (Supplementary Data S7, panel e).

In summary, information transmission at low TNF concentrations is mostly limited by molecular noise at the TNFR level, whereas at high TNF concentrations it is mostly limited by saturation of active IKK. As a result, the highest discernibility of adjacent TNF doses is observed at intermediate TNF doses: between 0.03 and 0.3 ng/ml.

### NF-κB network can integrate signals from different sources over time

**Figure 6:**
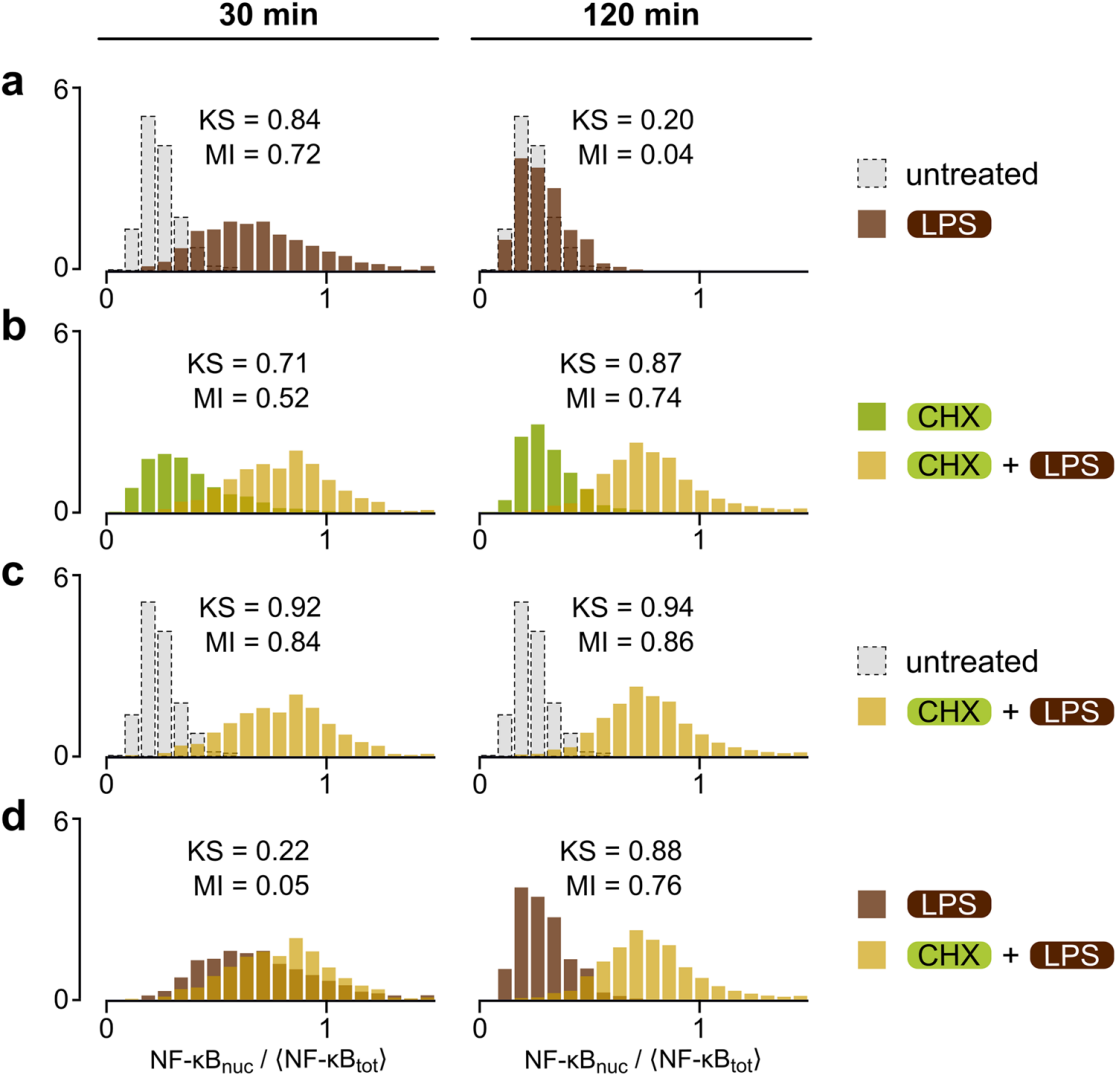
NF-κB system integrates information about inhibition of translation. MEFs were either stimulated with 1 μg/ml LPS for 30 or 120 min with or without 30-min pre-incubation with 5 μg/ml cycloheximide (CHX), or incubated with only CHX. Histograms (**a**–**d**) show NF-κB nuclear translocation based on confocal images of cells subjected to each type of stimulation. Between 500 and 700 cells were analysed for each histogram. NF-κB nuclear translocation is defined as the nuclear fluorescence normalized to the average whole-cell fluorescence (see Methods for details). Each plot contains overlying histograms for two stimulation types specified on the right, at the time point given at the top. Kolmogorov–Smirnov (KS) distances between the two samples (associated with two stimulation types as specified) and mutual information (MI) are given in each plot. See Supplementary Data S8 for corresponding uncropped immunostaining images.

In Fig. 6a we show that the capacity of the NF-κB system for distinguishing between the presence and absence of LPS is similar to that of TNF. Altogether, it confirms the earlier observation that within the first half hour the NF-κB system merely distinguishes between the presence and absence of a stimulus^1,2^. However, this is not true for longer times, during which the NF-κB network can integrate information from several sources. In Fig. 6 we show that when two time points are considered, 30 min and 120 min, cell responses are different for four types of treatment: absence of stimulation, stimulation with LPS, stimulation with CHX, and costimulation with CHX and LPS. Full frames of immunofluorescent confocal images showing MEFs stimulated with LPS, stimulated with CHX, or costimulated with CHX and LPS are provided in Supplementary Data S8. When comparing responses to LPS in the absence or presence of CHX costimulation (Fig. 6a versus Fig. 6b) we may notice that CHX costimulation somewhat reduces MI information at 30 min, but substantially increases information at 120 min. CHX blocks the synthesis of NF-κB inhibitors, IκBα and A20, allowing NF-κB to remain in the nucleus for at least two hours (Fig. 6c). As shown earlier in Fig. 1d, costimulation with CHX in addition to TNF or LPS resulted in markedly increased levels of IκBα and A20 mRNA at 3 hr/4 hr after TNF/LPS stimulation. Responses to the combined LPS + CHX stimulation, although almost indistinguishable from responses to pure LPS at 30 min, are clearly distinct at 120 min (Fig. 6d). This shows that LPS-stimulated cells are able to integrate signal from CHX over a longer time course.

## Discussion

In this study we analyzed how information about the strength of a stimulus is reduced when it propagates through subsequent steps of the NF-κB pathway. We quantified the NF-κB system’s ability to resolve consecutive TNF concentrations by KS distances between corresponding distributions of nuclear NF-κB. Using data-calibrated model we demonstrated that the ability to resolve small TNF concentrations is lost at the level of TNFR due to molecular noise associated with a very small number of activated receptors (Fig. 5). In contrast, high TNF concentrations, for which the molecular noise is negligible, are accurately resolved both at the level of TNFR and IKKK. However, ability to resolve high TNF concentrations is lost at the level of IKK and downstream components due to saturation of the response. As a result, at the level of NF-κB, the system’s ability to resolve stimulus levels is highest for intermediate concentrations, 0.03–1 ng/ml TNF. Based on the computational model we found that mutual information decreases with the strength of extrinsic noise. Importantly, noise at the TNF receptor level reduces information mainly upstream of NF-κB and not as much at the NF-κB level, at which information is controlled by the variability in total NF-κB levels. Considering NF-κB output only, one can see that increasing noise at the TNF receptor level reduces discernibility of intermediate TNF concentrations, but slightly increases discernibility of the highest concentrations. Therefore, extrinsic noise (here: variability in cellular sensitivity and variability in total NF-κB levels) reduces information available to the observer but can also widen the dynamic range allowing for better recognition of large stimulus concentrations.

Mutual information at the NF-κB level calculated from experimental data is about 0.8 bit. The model analysis indicates, however, that the transmitted information is somewhat higher. Discrepancy results from cytoplasmic interference, which causes that even unstimulated cells exhibit significant nuclear fluorescence. Based on the model we estimated the magnitude of this effect and found that the mutual information is close to 1 bit. This is in agreement with earlier findings by Cheong *et al*.^1^ and Selimkhanov *et al*.^2^, and indicates that by generating or not the first nuclear NF-κB pulse the cell recognizes, respectively, the presence or absence of stimulus, rather than senses strength of the stimulus. Information transmitted to a transcription factor can be further processed by activating target genes^20,21^. After TNF stimulation, the expression of early NF-κB-responsive genes, calculated per responding cell, is independent of TNF concentration^13^, which indicates all-or-nothing early gene activation in single cells responding to TNF. Recently we demonstrated that for LPS stimulation even the amplitude of NF-κB translocation is only weakly dependent on the concentration: rather, for smaller concentrations the response probability is lower and the response is delayed^19^. These findings suggest that in early responses cells exhibit stochastic robustness, which on one hand allows a single cell to either respond or ignore the stimuli, but on the other hand leads to synchronized early-gene activation when a cell decides to respond^22^.

The reduction of input information to a single bit may suggest that the NF-κB system has not evolved to accurately transmit information, but rather constitutes a decision-making module, which based on input chooses whether or not to activate transcription of the specific early genes. Multiple nonlinear transmission elements that are present in the NF-κB pathway (out of which only few are included in the model) facilitate digitization of the response. These elements include trimerization of TNFR^23^, double phosphorylation of IKK subunit IKKβ^24^, double phosphorylation of IκBα at Ser32 and Ser36 by active IKK^25^, which is required for IκBα ubiquitination and proteasomal degradation, and multiple NF-κB binding sites on early gene promoters^26^. These elements are capable of increasing the response to signals exceeding a certain threshold. Additionally, negative feedbacks mediated by NF-κB inhibitors limit NF-κB activation temporally. TNF stimulation leads to a peak of NF-κB activity, which may be followed by subsequent pulses when the signal persists^27^. The subsequent NF-κB activity pulses are well pronounced when TNF also appears in the form of pulses with a sufficiently long period (exceeding 60 min)^9,28,29^. Nonlinear transmission elements combined with negative feedbacks result in a response that is digitized into pulses of NF-κB activity and transcription bursts of early genes^29^. The same mechanism was observed for other pathways. In the MAPK pathway, positive feedback nested within the negative feedback loop results in recurrent pulses of ERK activity^30,31^. In the context of the p53 pathway, recurrent waves of AKT phosphorylation^32^ are transcoded into pulses of p53 and Mdm2 levels observed over days in MCF7 cells upon DNA damage^33^. Information loss can be beneficial when it allows for cellular decision making. Intuitively this is the case in bistable systems, when the signal strength and noise dictate probabilities of predefined responses. Because the decision making has a stochastic component, for given stimuli cell population can split into two (or more) distinct subpopulations^34^. In such a case the response is analog at the population level (with fraction of cells in a given subpopulation governed by the strength of stimuli) and digital at single-cell level.

In oscillatory systems, disruption of the negative feedback qualitatively changes the character of the response. In the context of p53, we and others proposed that the disruption of the p53–Mdm2 negative feedback that follows PTEN accumulation terminates p53 oscillations, leading to an abrupt increase of p53 level and apoptosis^3,35^. Here, we show that disruption of A20- and IκBα-mediated feedbacks by incubation with CHX leads to prolonged NF-κB activation in responses to TNF or LPS. At 15 or 30 min, responses of cells stimulated by a high concentration of TNF and TNF + CHX are similar, but at 3 hr cells incubated with CHX prior to administration of TNF exhibit much higher NF-κB activity and highly increased levels of IκBα and A20 transcripts. Similarly LPS and LPS + CHX stimulated cells exhibit similar responses at 30 min (KS = 0.22), but clearly distinct responses at 120 min (KS = 0.88) which is also visible at the level of gene expression. This shows that the NF-κB system not only transmits signals from receptors, but also is able to integrate other signals over time, producing qualitatively different responses. As inhibition of IκBα and A20 protein synthesis highly increases levels of the IκBα and A20 transcripts, one could expect that it exerts even stronger effect on the late NF-κB-responsive genes, which require prolonged presence of NF-κB in the nucleus^36^. The examples of CHX + TNF and LPS + CHX costimulation, although somewhat contrived cases of mixed physiological and non-physiological stimulation, are important because inhibition of protein synthesis can result from activation of the PKR–eIF2α pathway in response to poly(I:C) or viral stimulation^37^, and both of them lead to NF-κB activation^38^. The discussed mechanism would thus allow to distinguish between LPS (or bacterial) and poly(I:C) (or viral) stimulation. As suggested in Fig. 6, the former leads to the pulse-like NF-κB activation, whereas the latter would lead to switch-like prolonged activation, as observed by Rand *et al*.^39^ in response to viral stimulation.

Intrinsic, molecular noise and response saturation limit the information transmission to NF-κB for low and high stimulation, respectively, thus creating a window of system sensitivity between 0.03 and 1 ng/ml, and limiting information transmitted within first 30 min to 1 bit. Having analyzed how information is reduced as the signal is propagated through subsequent steps of the NF-κB signaling pathway, we conclude that the system constitutes a decision-making module that digitizes the signal into pulses of NF-κB activity, rather than an accurate information transmitting channel. Over longer periods of time, the system is capable of integrating signals that block the synthesis of NF-κB inhibitors and produces qualitatively different, switch-like responses to CHX + TNF, or CHX + LPS costimulation, in contrast to pulse-like responses to stimulation with sole TNF or LPS. Based on these results we hypothesize the NF-κB system has several predefined responses, associated with activation of particular groups of genes, and the aim of the transduction pathway is to convert incoming signals into these responses. Molecular noise underlies the probabilistic character of this conversion, whereas the presence of nonlinear regulatory elements reduces the chance that the system will exhibit responses from beyond the predefined repertoire.

## Methods

### Cell culture and compounds

Experiments were performed on wild-type MEFs derived in the Brasier Laboratory^40^. Line derivation was conducted under vertebrate animal protocols approved by the UTMB Animal Care and Utilization Committee. The cell line was routinely tested against mycoplasma contamination by DAPI staining and PCR. Cells were cultured in adherent conditions on tissue culture-treated dishes (Falcon) in complete Dulbecco’s modified medium (DMEM) with 4.5 g/l of D-glucose and 0.1 mM L-glutamine (ThermoFisher Scientific), with addition of 10% fetal bovine serum (ThermoFisher Scientific) and 100 mg/ml penicillin/streptomycin mix (Sigma-Aldrich). The culture was maintained in a conditioned incubator at 37 °C and 5% CO_2_. For stimulation, cells were seeded on multi-well plates or coverslips and allowed to adhere overnight at 37 °C. Mouse recombinant TNF, LPS from Escherichia coli 0111:B4 (purified by ion-exchange chromatography) and CHX were purchased from Sigma-Aldrich. LPS was solubilized in a bath sonicator for 15 min and vortexed vigorously for additional 1 min prior to adding to the cells. CHX was added to the cells at a final concentration of 5 μg/ml 30 minutes prior to the addition of TNF or LPS.

### Immunostaining

After stimulation, cells were subjected to the following consecutive steps: fixation with 4% formaldehyde for 20 min, neutralization of formaldehyde groups with 50 mM NH_4_Cl for 10 min, membrane permeabilization with 0.1% Trition X-100 for 5 min and blocking with 5% BSA in PBS for 1 h. Primary antibodies were added to the cells in 1:1000 dilution in 5% BSA for 90 min, and the same was repeated for secondary antibodies. Nuclei were then stained with 200 ng/ml DAPI in PBS for 10 min. The coverslips were mounted onto microscope slides with Mowiol (Sigma-Aldrich) and observed using Leica TCS SP5 X confocal microscope with Leica Application Suite AF software.

### Microscopic image analysis

*Image analysis*. Confocal images obtained from immunostaining were analyzed using our in-house software. Based on DAPI nuclear staining, nuclear contours were automatically detected. Contours that marked nuclear regions inaccurately during automatic detection were then drawn manually, and contours of nuclei that were partially out of frame, overlapping, mitotic or otherwise misshapen were manually excluded. For background noise correction, in each frame at least 3 background intensity-defining regions, which did not contain any cells, were indicated manually. Background intensity (in each channel) was quantified as the average fluorescence in these regions. The resulting quantifications were analyzed with auxiliary Matlab scripts, which provided estimates of the magnitude of nuclear translocation. The applied image analysis method is motivated by the relative ease of detection of nuclear contours (based on DAPI staining) and inability to accurately determine cytoplasmic contours in an automated way.

*Quantification of NF-κB nuclear fraction*. Nuclear NF-κB fraction in *i* ^th^ cell was calculated as:

10 $${{\rm{NFkB}}}_{{\rm{nuc}}}/{{\rm{NFkB}}}_{{\rm{total}}}(i)=\frac{{\tilde{I}}_{{{\rm{n}}}_{i}}^{{\rm{NFkB}}}}{{\tilde{I}}_{\ast }^{{\rm{NFkB}}}}\frac{{\tilde{I}}_{\ast }^{{\rm{DAPI}}}}{{\tilde{I}}_{{{\rm{n}}}_{i}}^{{\rm{DAPI}}}},$$

where ${\tilde{I}}_{{{\rm{n}}}_{i}}^{{\rm{NFkB}}}$ and ${\tilde{I}}_{{{\rm{n}}}_{i}}^{{\rm{DAPI}}}$ are the background-corrected fluorescence intensities within the *i* ^th^ nuclear contour, whereas ${\tilde{I}}_{\ast }^{{\rm{NFkB}}}$ and ${\tilde{I}}_{\ast }^{{\rm{DAPI}}}$ are the background-corrected fluorescence intensities of the whole confocal image area, in the NF-κB and DAPI channel, respectively. The normalization using DAPI staining corrects possible errors resulting from out-of-focus cell displacements, as intensity of displaced cells registers weakly in both the NF-κB and DAPI channels.

Background-corrected fluorescence intensity in the *i* ^th^ nucleus, ${\tilde{I}}_{{{\rm{n}}}_{i}}$, in a given channel is defined as the difference between the fluorescence intensity in the *i* ^th^ nucleus, ${I}_{{{\rm{n}}}_{i}}$, and the average background pixel intensity $\langle {p}_{{\rm{bg}}}\rangle$ in that channel multiplied by the *i* ^*th*^ nuclear contour surface area, ${S}_{{{\rm{n}}}_{i}}$:

11 $${\tilde{I}}_{{{\rm{n}}}_{i}}^{{\rm{NFkB}}}={I}_{{{\rm{n}}}_{i}}^{{\rm{NFkB}}}-{S}_{{{\rm{n}}}_{i}}^{{\rm{NFkB}}}\times \langle {p}_{{\rm{bg}}}^{{\rm{NFkB}}}\rangle ,$$

12 $${\tilde{I}}_{{{\rm{n}}}_{i}}^{{\rm{DAPI}}}={I}_{{{\rm{n}}}_{i}}^{{\rm{DAPI}}}-{S}_{{{\rm{n}}}_{i}}^{{\rm{DAPI}}}\times \langle {p}_{{\rm{bg}}}^{{\rm{DAPI}}}\rangle .$$

Analogically, background-corrected fluorescent intensity of the image, ${\tilde{I}}_{\ast }$, in a given channel is defined as the difference between the fluorescent intensity of the image, ${I}_{\ast }$ and the average background pixel intensity $\langle {p}_{{\rm{bg}}}\rangle$ in that channel multiplied by the whole image area, ${S}_{\ast }$:

13 $${\tilde{I}}_{\ast }^{{\rm{NFkB}}}={I}_{\ast }^{{\rm{NFkB}}}-{S}_{\ast }^{{\rm{NFkB}}}\times \langle {p}_{{\rm{bg}}}^{{\rm{NFkB}}}\rangle ,$$

14 $${\tilde{I}}_{\ast }^{{\rm{DAPI}}}={I}_{\ast }^{{\rm{DAPI}}}-{S}_{\ast }^{{\rm{DAPI}}}\times \langle {p}_{{\rm{bg}}}^{{\rm{DAPI}}}\rangle .$$

### Gene expression analysis

#### RNA extraction and reverse transcription

Cells were seeded on 12-well plates one day before the experiment at a density of around 100,000 per well. After stimulation, cells were washed once with PBS and total RNA was isolated using PureLink RNA Mini Kit (ThermoFisher Scientific) according to the manufacturer’s instructions. Concentration of isolated RNA was determined by measuring UV absorbance of samples at 1:100 dilution at 260 nm and 280 nm with a Multiskan GO Microplate Spectrophotometer (ThermoFisher Scientific). Reverse transcription with random primers was performed using High Capacity cDNA Reverse Transcription Kit (ThermoFisher Scientific). The process was conducted in a Mastercycler Gradient thermal cycler (Eppendorf) with the following settings: 10 min/25 °C, 120 min/37 °C, and 5 min/85 °C.

#### Digital PCR

Digital PCR was performed for the IκBα and A20 genes using QuantStudio 3D system (Life Technologies). Prepared samples were loaded into QuantStudio 3D Digital PCR Chip and thermocycled using the ProFlex PCR System (ThermoFisher Scientific) according to the manufacturer’s guidelines. Chip analysis was performed using QuantStudio 3D Digital PCR Instrument and Analysis Suite cloud software. Measurements of the amount of glyceraldehyde 3-phosphate dehydrogenase (GAPDH) mRNA were used as a normalization reference for input sample quantity.

### Western blotting

#### Cell-fractionation

Cells were seeded on a 100-mm tissue culture-treated dishes, at a density of 1,000,000/dish, and incubated overnight. After stimulation, cells were placed on ice, washed with ice-cold PBS, scraped from the dish in PBS and centrifuged (4 °C, 100 × g, 5 min). Cell pellet was then suspended in 1.5 ml of hypotonic cytoplasmic fraction buffer (20 mM HEPES pH 8.0, 0.2% IGEPAL CA-630, 1 mM EDTA, 1 mM DTT, protease and phosphatase inhibitor cocktail, as above) and incubated on ice for 10 min with occasional shaking. After centrifugation (4 °C, 1700 × g, 5 min), supernatant was set aside and treated as the cytoplasmic fraction; pellet was washed in the same buffer and recentrifuged, and supernatant was discarded. Remaining pellet was suspended in 150 μl of nuclear fraction buffer (20 mM HEPES pH 8, 420 mM NaCl, 20% glycerol, 1 mM EDTA, 1 mM DTT, protein and phosphatase inhibitors, as above), incubated on ice for 30 min with occasional mixing and then centrifuged at 4 °C, 10,000 × g, 10 min. Supernatant containing nuclear fraction was transferred to a fresh tube and left for further processing.

#### SDS-PAGE and Western blot

Protein concentrations in cell lysates were determined using the Bradford method against a BSA standard. After precipitation and washing, proteins were resuspended in standard Laemmli sample buffer containing 10 mM DTT and boiled at 95 °C for 10 min. Equal amounts of each protein sample was loaded onto 10% polyacrylamide gel and SDS-PAGE was performed with Mini-PROTEAN Tetra System (Bio-Rad). Proteins were transferred to nitrocellulose membrane using wet electrotransfer in the Mini-PROTEAN apparatus and blocked for 60 min with 5% non-fat dry milk. Membranes were incubated at 4 °C overnight with anti-p65 D14E12 (CST) or anti-HDAC-1 (Santa Cruz Biotechnology) primary antibody. After washing with TBST, membranes were incubated with secondary antibodies conjugated with horseradish peroxidase (goat anti-rabbit and anti-mouse immunoglobulins/HRP, Dako) for 60 min at room temperature. Specific proteins were detected in the dark room on a medical X-ray film using Clarity Western ECL system (Bio-Rad). Densitometric quantification of protein bands was performed with ImageJ software, using normalization against HDAC1.

### Computational model and numerical simulations

Stochastic numerical simulations according to the Gillespie algorithm^41^ were performed in BioNetGen ^42,43^ using the NF-κB computational model described by Korwek *et al*.,^11^ with two modifications introduced to obtain better fit to the experimental data:

1. We assumed that on average there are 10^5^ NF-κB molecules in the translocatable pool and 0.5 × 10^5^ NF-κB molecules in the inert pool.
2. We assumed that the median number of TNFR per cell is equal 2 × 10^3^.

We provide the BioNetGen language-encoded model code in Supplementary Dataset S1. Before running each stochastic simulation, the number of NF-κB molecules in the translocatable and inert pools is drawn at random from the experimental distribution shown in Fig. 1c (with assumed average 10^5^ molecules for the translocatable pool and 0.5 × 10^5^ for the inert pool) and inserted into the BNGL file using in-house scripts. The number of receptors is drawn at random using log-normal distribution *LnN*(µ, σ^2^) with constant median e^µ^ = 2 × 10^3^ and one of four values of σ: 0, 0.3, 1, 3. Simulations without extrinsic noise are performed by setting σ = 0, and by setting number of NF-κB molecules in the translocatable pool and 0.5 × 10^5^ NF-κB molecules in the inert pool. In each case we perform 10,000 stochastic simulations mimicking samples of 10,000 cells.

To compare numerical simulations with experimental data we calculate normalized distribution of nuclear NF-κB, ${({{\rm{NFkB}}}_{{\rm{nuc}}})}_{{\rm{norm}}}^{{\rm{CI}}}$, taking into account: 1) inert NF-κB pool, 2) cytoplasmic interference (as estimated in Results, 0.24 of the cytoplasmic fluorescence registers as the nuclear):

15 $${({{\rm{NFkB}}}_{{\rm{nuc}}})}_{{\rm{norm}}}^{{\rm{CI}}}=[{N}_{{\rm{nuc}}}+0.24({N}_{{\rm{cyt}}}+{N}_{{\rm{inert}}})]\times {(1.5\times {10}^{5})}^{-1},$$

where *N*_nuc_ and *N*_cyt_ denote nuclear and cytoplasmic NF-κB obtained from stochastic simulations, whereas *N*_inert_ is the amount of inert NF-κB. Whenever we refer to simulations “with cytoplasmic interference”, we refer to the above normalization.

To analyze the “unmasked” signal transmission, we calculate normalized distribution of nuclear NF-κB, ${({{\rm{NFkB}}}_{{\rm{nuc}}})}_{{\rm{norm}}}$, without accounting for cytoplasmic interference:

16 $${({{\rm{NFkB}}}_{{\rm{nuc}}})}_{{\rm{norm}}}={N}_{{\rm{nuc}}}\times {(1.5\times {10}^{5})}^{-1}.$$

### Estimation of the upper bound of mutual information

The mutual information (MI) was calculated according to the method of Kraskov *et al*.^17^. The maximization of MI with respect to the set of eight *a priori* probabilities was performed using a steepest-ascent method. The accuracy of MI estimation and its maximization was performed by drawing random samples from eight overlapping Gaussian distributions. MI and its upper bound were calculated based on the drawn samples and compared with MI calculated directly from the Gaussian distributions using an auxiliary Mathematica code. The chosen Gaussian distributions have increasing means and increasing variances and resemble experimental distributions of nuclear NF-κB from experiments with consecutive TNF doses. Sample sizes were equal to the numbers of cells analyzed in experiment (30-min time point) for each TNF dose. In this way, the inaccuracy of the estimation of the upper bound of MI based on experimental data was found to be about 2–3%. For each TNF dose, 10,000 stochastic simulations were performed and thus the reported estimates of MI are expected to have a smaller error. Python and Mathematica codes are provided in Supplementary Dataset S2.

## Electronic supplementary material

Supplementary Information

Dataset 1

Dataset 2
