# Supplementary material for: Information processing in the NF-κB pathway
Source: Sci Rep. 2017 Nov 21;7:15926. doi: 10.1038/s41598-017-16166-y (PMC5698458; doi:10.1038/s41598-017-16166-y)
Supplement: Supplementary file 1 — Supplementary Information [file 41598_2017_16166_MOESM1_ESM.pdf]

Information processing in the NF-κB pathway

K. TUDELSKA, J. MARKIEWICZ, M. KOCHAŃCZYK, M. CZERKIES, W. PRUS, Z. KORWEK,
A. ABDI, S. BŁOŃSKI, B. KAŹMIERCZAK AND T. LIPNIACKI

Supplementary Information

Supplementary Data S1. Full frames of immunofluorescent confocal images showing MEFs stimulated with TNF or costimulated with CHX and TNF.

Supplementary Data S2. Full-length Western blots of nuclear RelA in response to TNF.

Supplementary Data S3. Full frames of immunofluorescent confocal images showing MEFs stimulated with eight concentrations of TNF.

Supplementary Data S4. Kolmogorov–Smirnov distances and mutual information of NF-κB$_{nuc}$ for all pairs of TNF concentrations.

Supplementary Data S5. The effect of cytoplasmic interference on Kolmogorov–Smirnov distances analyzed based on numerical simulations.

Supplementary Data S6. Histograms of pathway components after stimulation with 8 TNF concentrations.

Supplementary Data S7. The effect of extrinsic noise on Kolmogorov–Smirnov distances and mutual information.

Supplementary Data S8. Full frames of immunofluorescent confocal images showing MEFs stimulated with LPS or costimulated with CHX and LPS.

Two supplementary datasets that feature the article are provided separately:

Supplementary Dataset S1. Model implementation in BioNetGen language.

Supplementary Dataset S2. Python implementation of the Kraskov algorithm for estimating mutual information.

Supplementary Data S1.

Full frames of immunofluorescent confocal images showing MEFs stimulated with TNF or costimulated with CHX and TNF.

Immunofluorescent confocal images whose fragments are shown in Fig 1b. MEFs were stimulated with 10 ng/ml TNF in the absence or presence of 5 μg/ml CHX. In the CHX+TNF costimulation experiment, incubation with 5 μg/ml CHX started 30 min before TNF stimulation. Cells were fixed and stained with antibodies for RelA (a subunit of NF-κB; green) and for IκBα (red) at 0 (untreated), 15, 30, and 180 min after TNF stimulation.

Supplementary Data S1.

0 min (control)

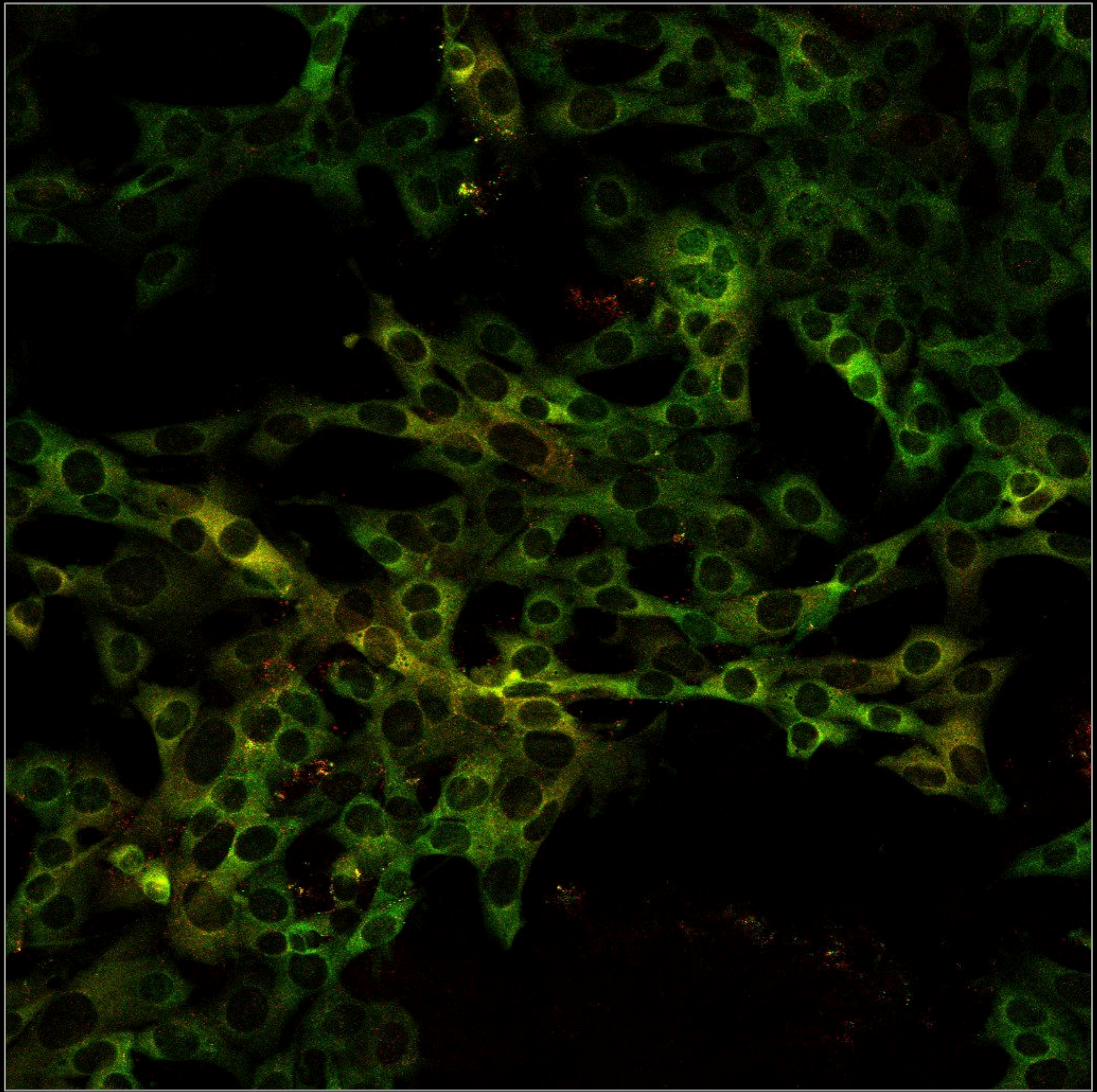

RelA / IκBα

Supplementary Data S1.

15 min TNF 10 ng/ml

RelA / IκBα

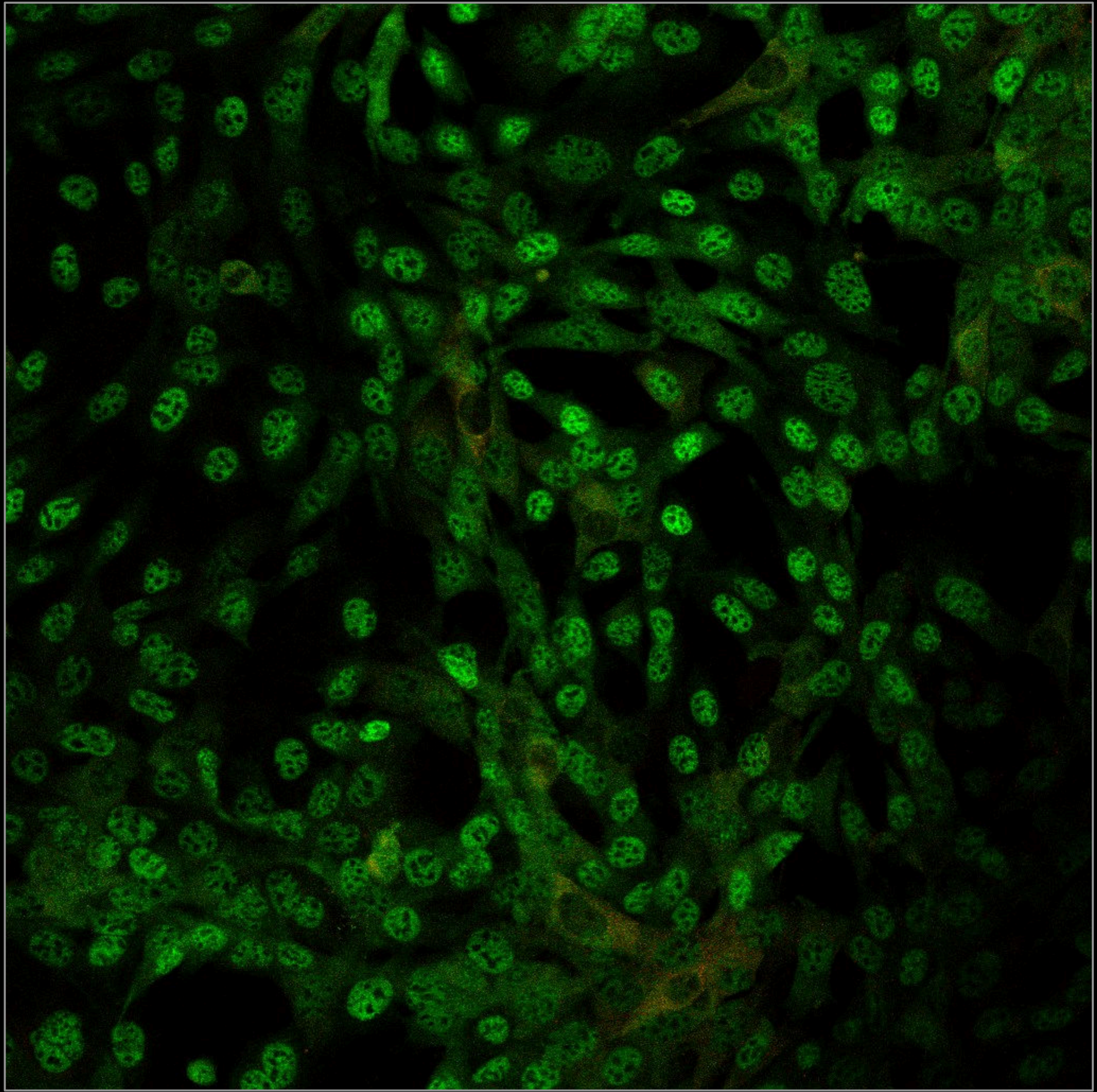

Supplementary Data S1.

30 min TNF 10 ng/ml

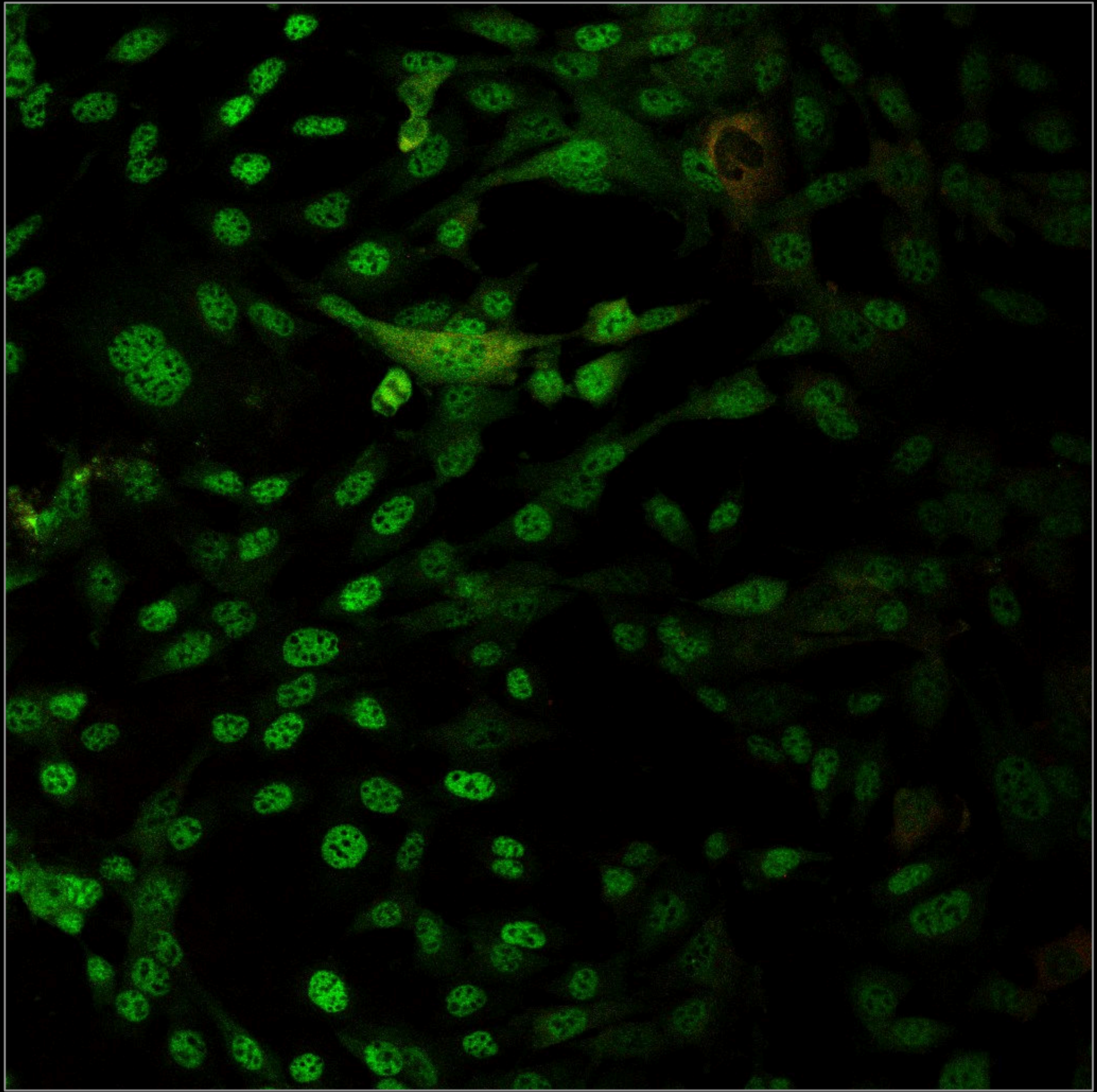

RelA / IκBα

Supplementary Data S1.

180 min TNF 10 ng/ml

RelA / IκBα

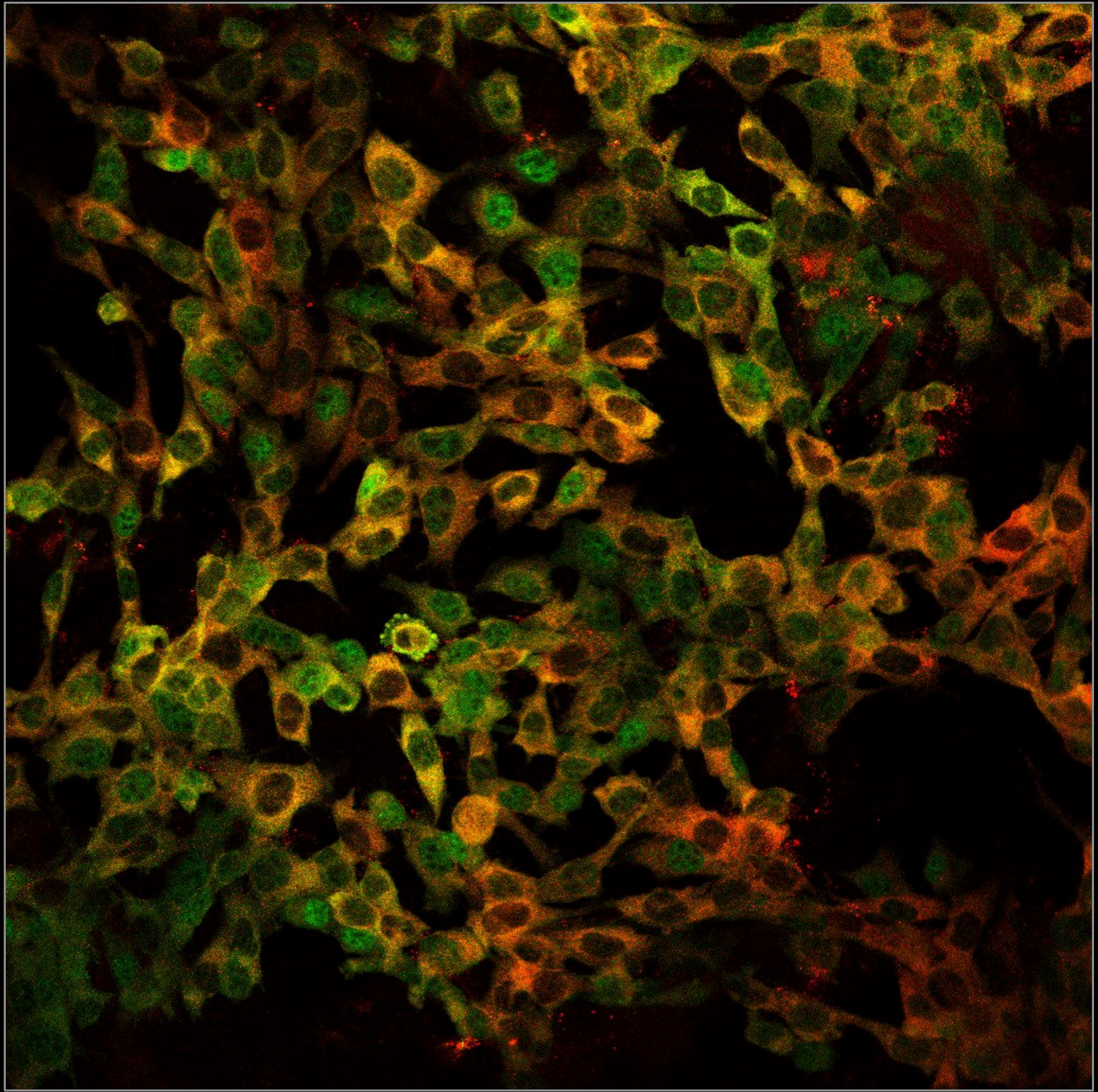

Supplementary Data S1.

30 min CHX 5 μg/ml

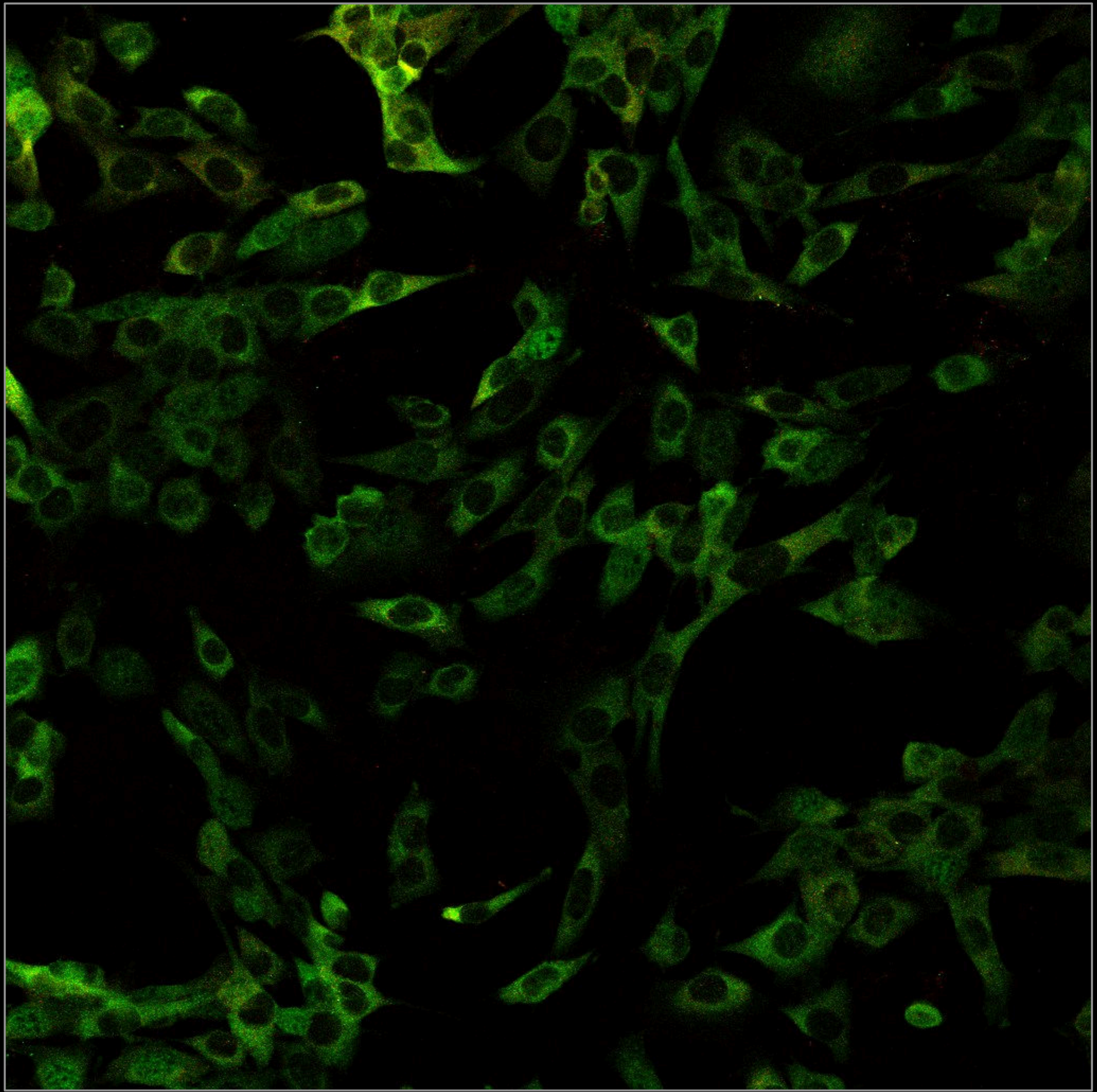

RelA / IκBα

Supplementary Data S1.

CHX+TNF costimulation:
5 μg/ml CHX added
30 min before TNF

15 min TNF 10 ng/ml

RelA / IκBα

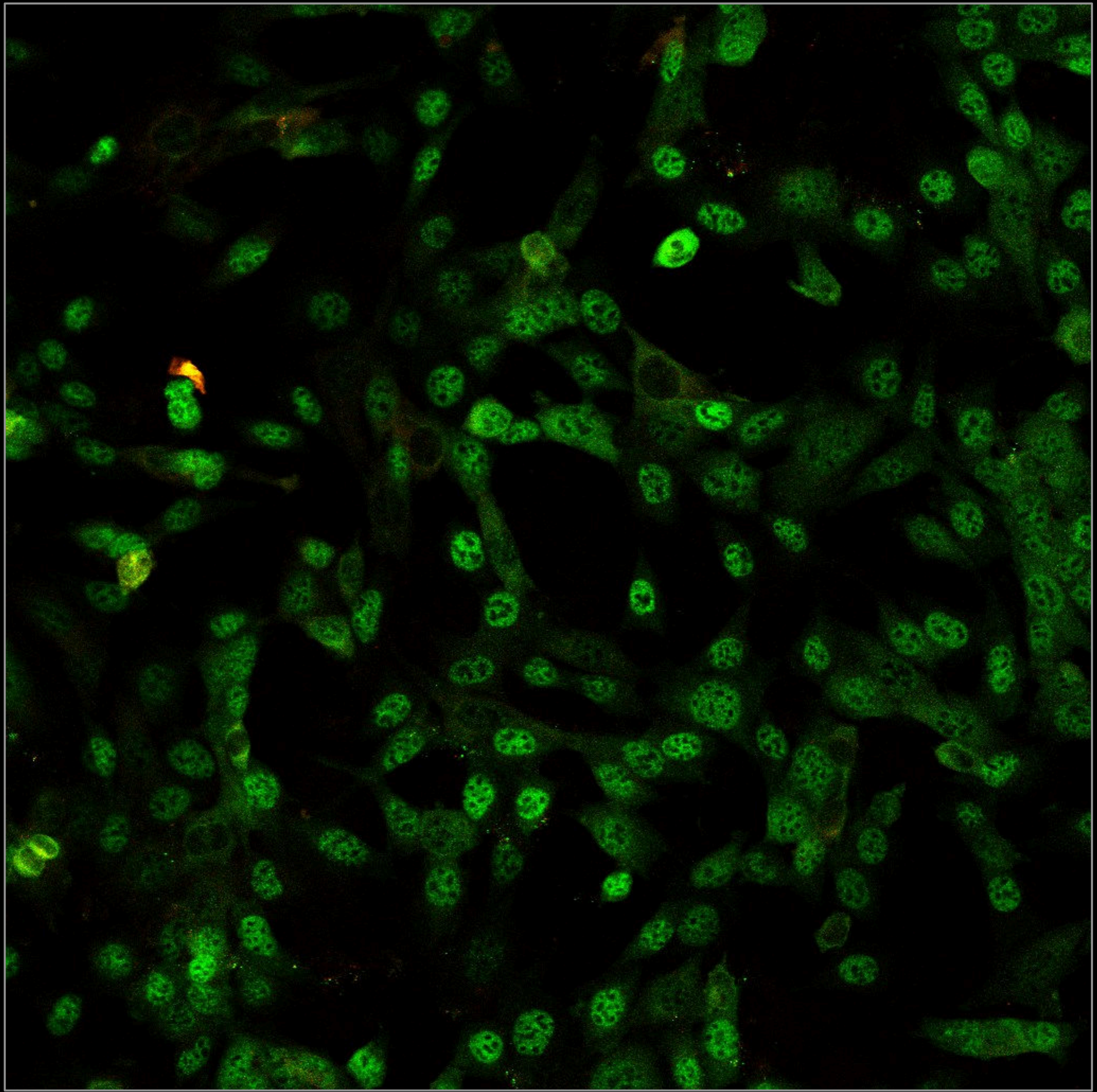

Supplementary Data S1.

CHX+TNF costimulation:
5 μg/ml CHX added
30 min before TNF

30 min TNF 10 ng/ml

RelA / IκBα

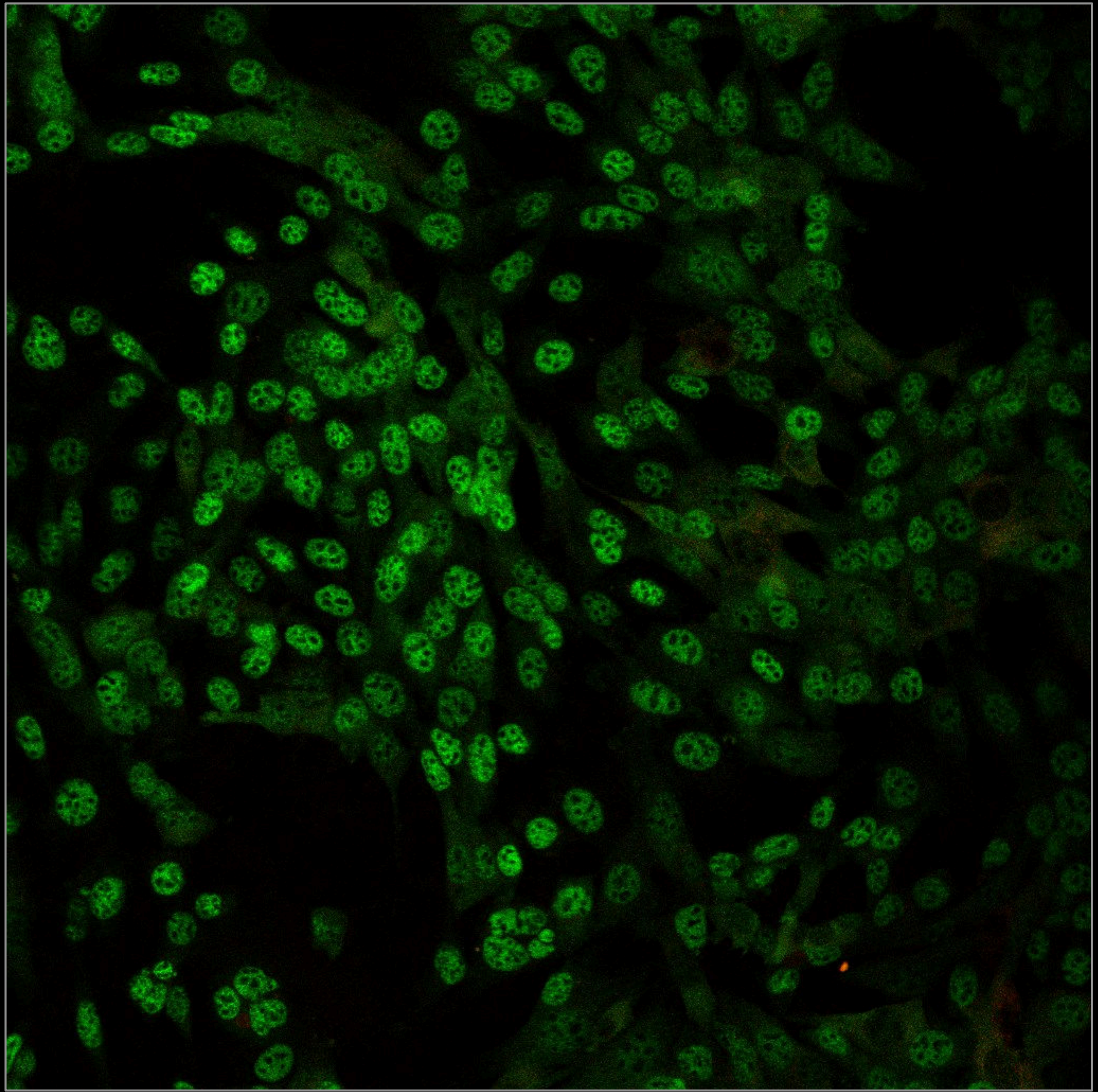

Supplementary Data S1.

CHX+TNF costimulation:
5 μg/ml CHX added
30 min before TNF

180 min TNF 10 ng/ml

RelA / IκBα

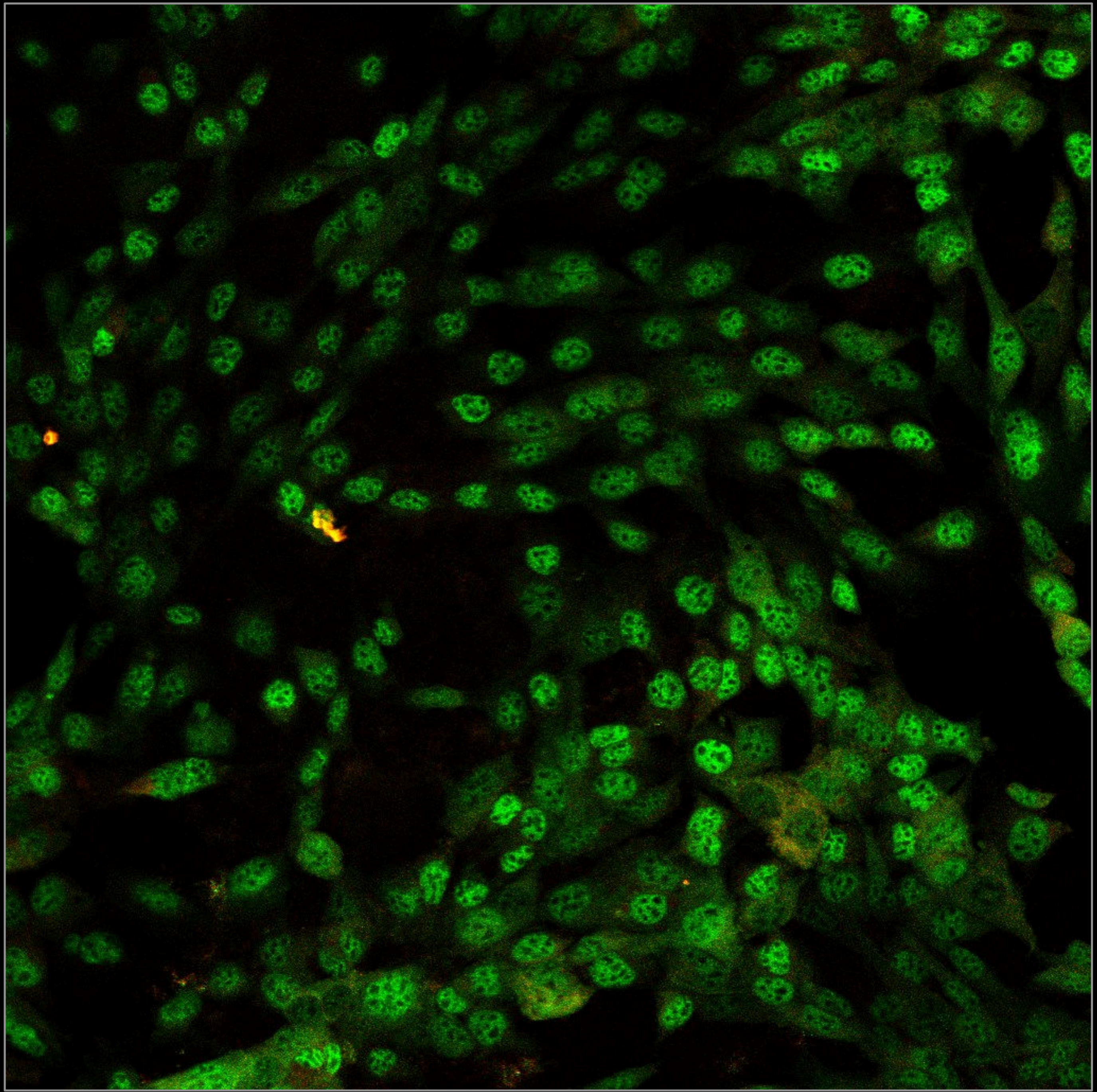

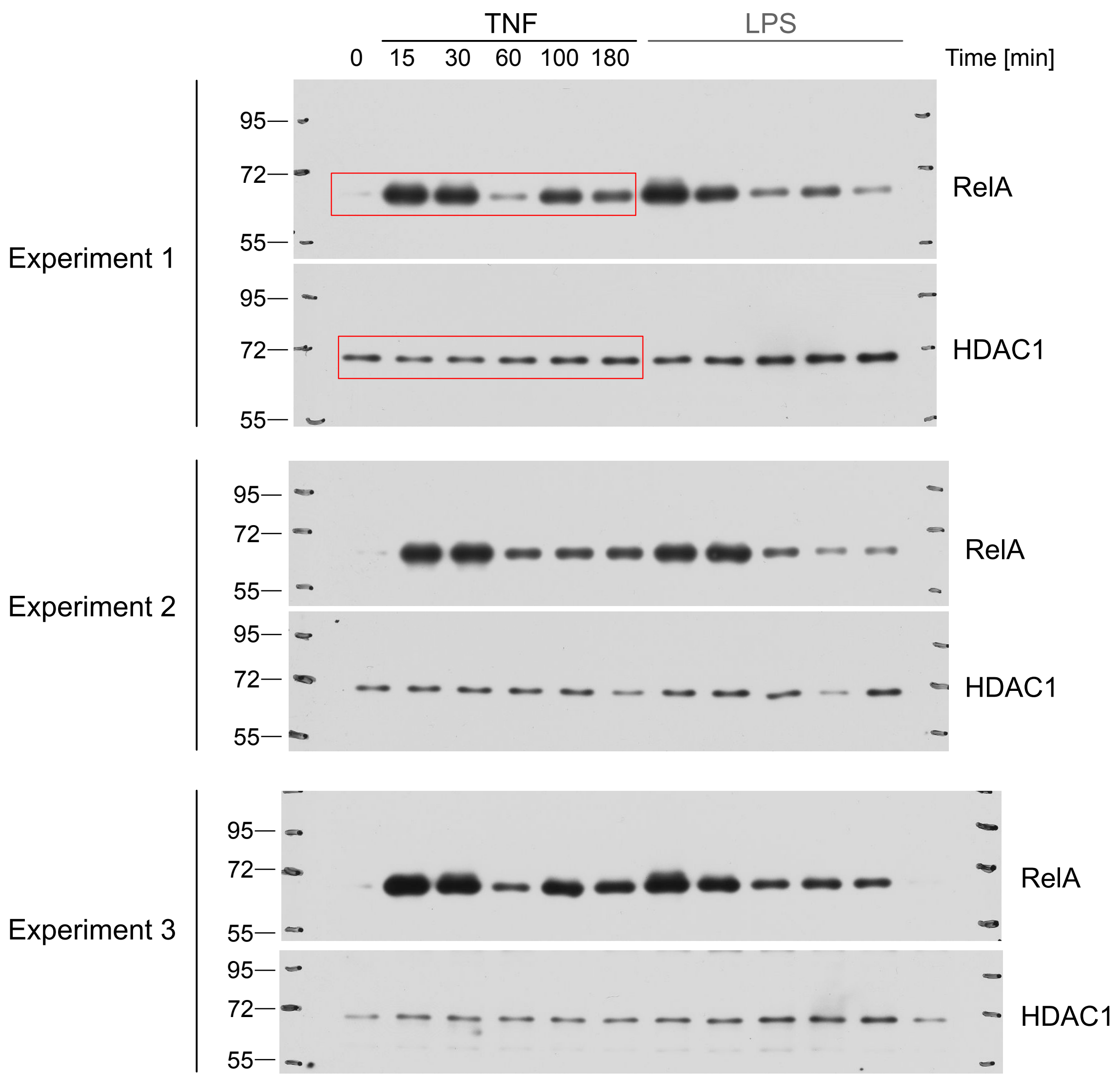

Supplementary Data S2.

Full-length Western blots of nuclear RelA in response to TNF.

All three blots were used for densitometric quantification which is summarized as a bar plot in Fig. 1e in the main text. Fragments of blots labeled 'Experiment 1' are shown in Fig. 1e in the main text.

Supplementary Data S3.

Full frames of immunofluorescent confocal images showing MEFs stimulated with eight concentrations of TNF.

Immunofluorescent confocal images whose fragments are shown in Fig 3. MEFs were stimulated with 0 (untreated), 0.01, 0.03, 0.1, 0.3, 1, 3 or 10 ng/ml TNF. Cells were then fixed and stained with antibodies for RelA (a subunit of NF-κB; green) and for IκBα (red) at 15 or 30 min after TNF stimulation.

Supplementary Data S3.

MEF + TNF **0.00** ng/ml (untreated)

15 min

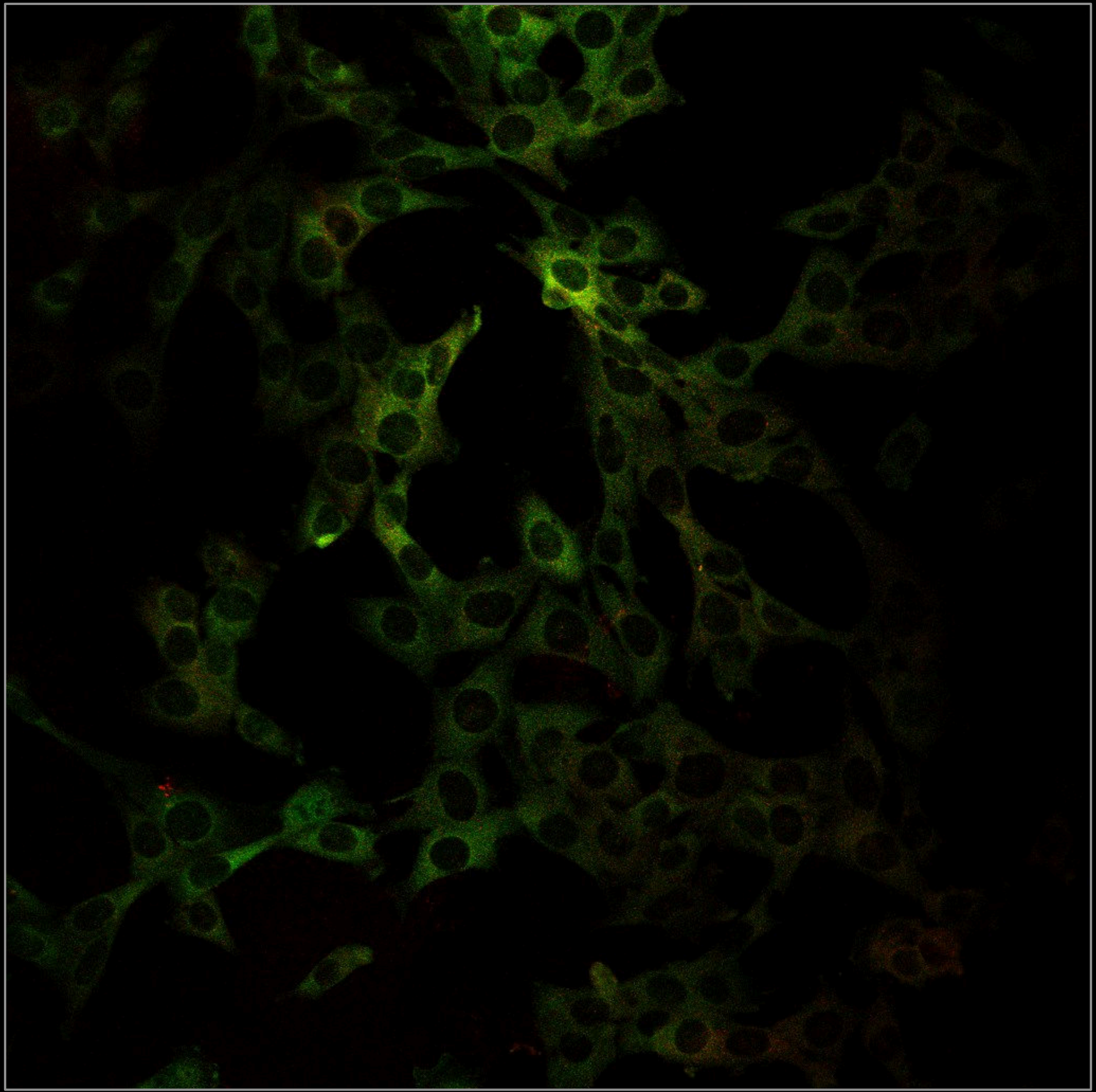

RelA / IκBα

30 min

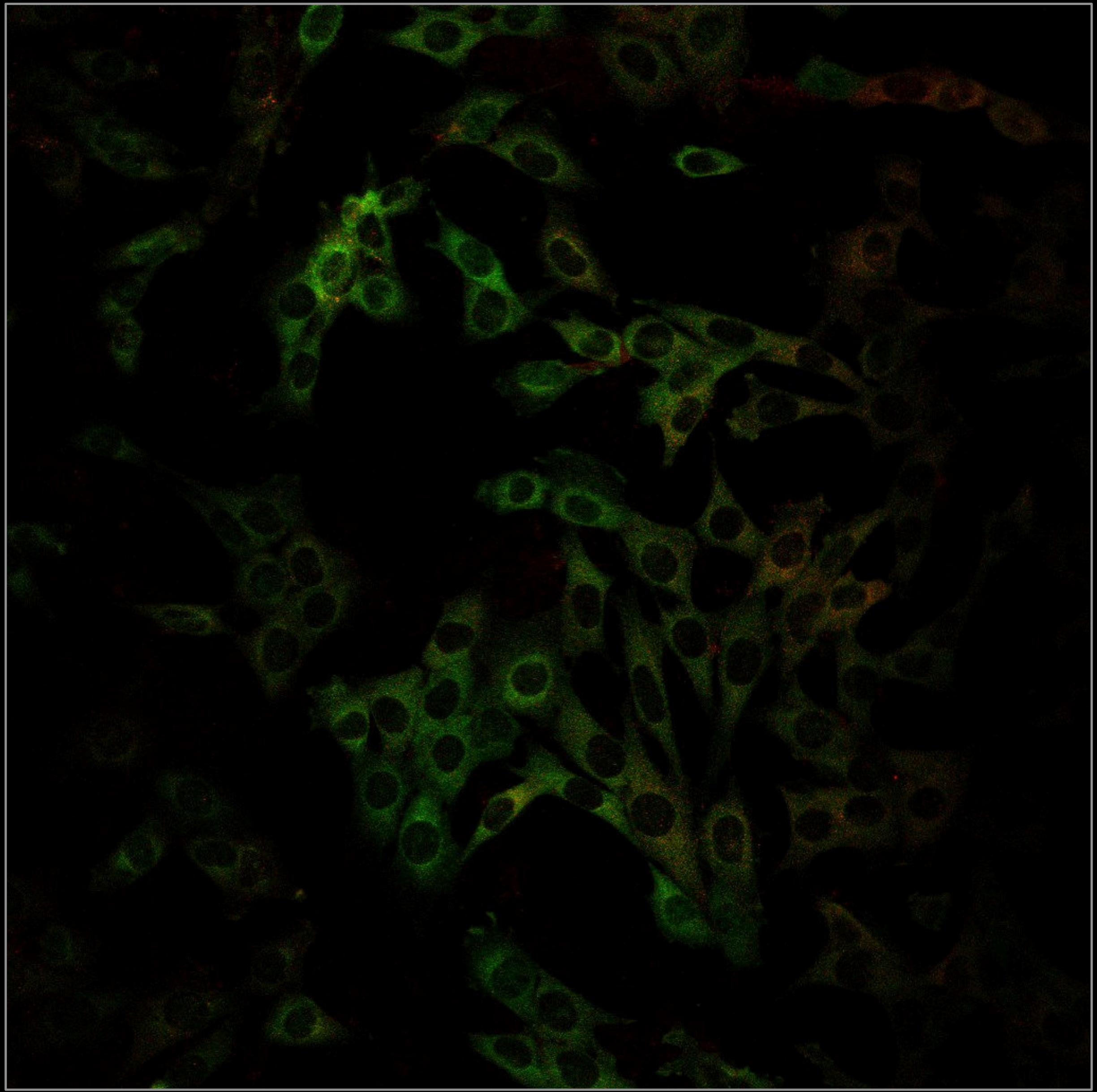

RelA / IκBα

Supplementary Data S3.

MEF + TNF **0.01** ng/ml

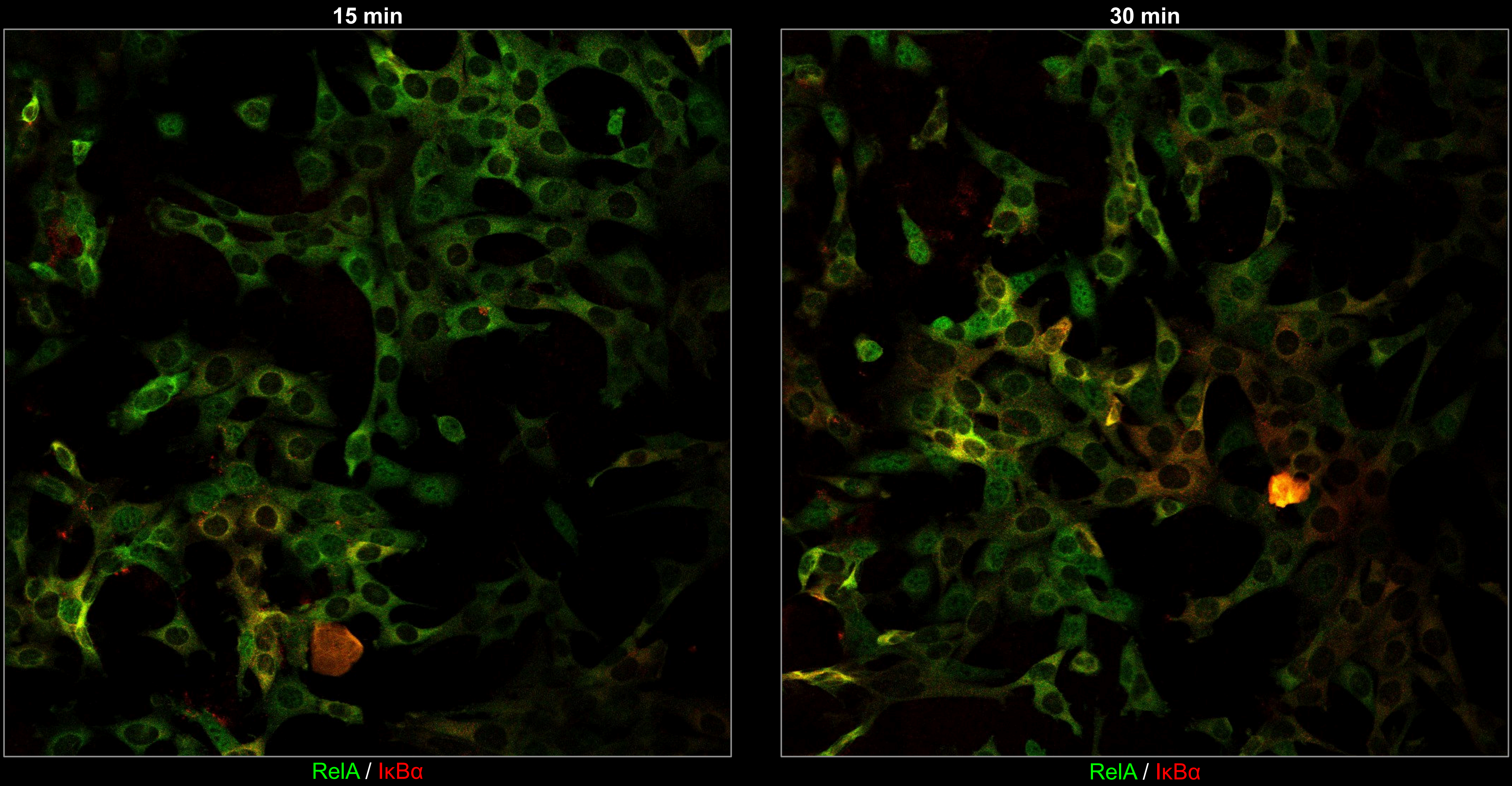

Supplementary Data S3.

MEF + TNF **0.03** ng/ml

15 min

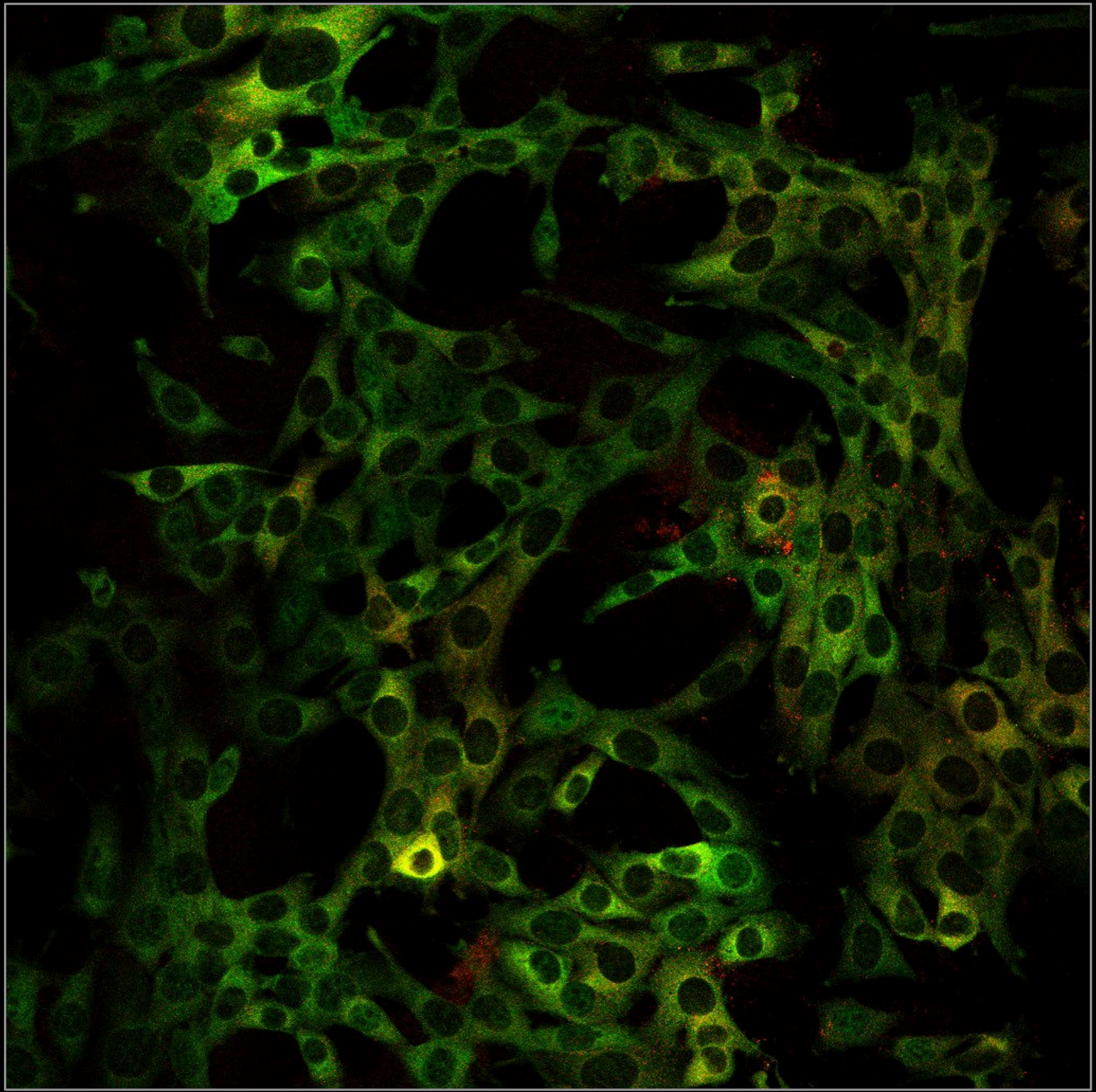

RelA / IκBα

30 min

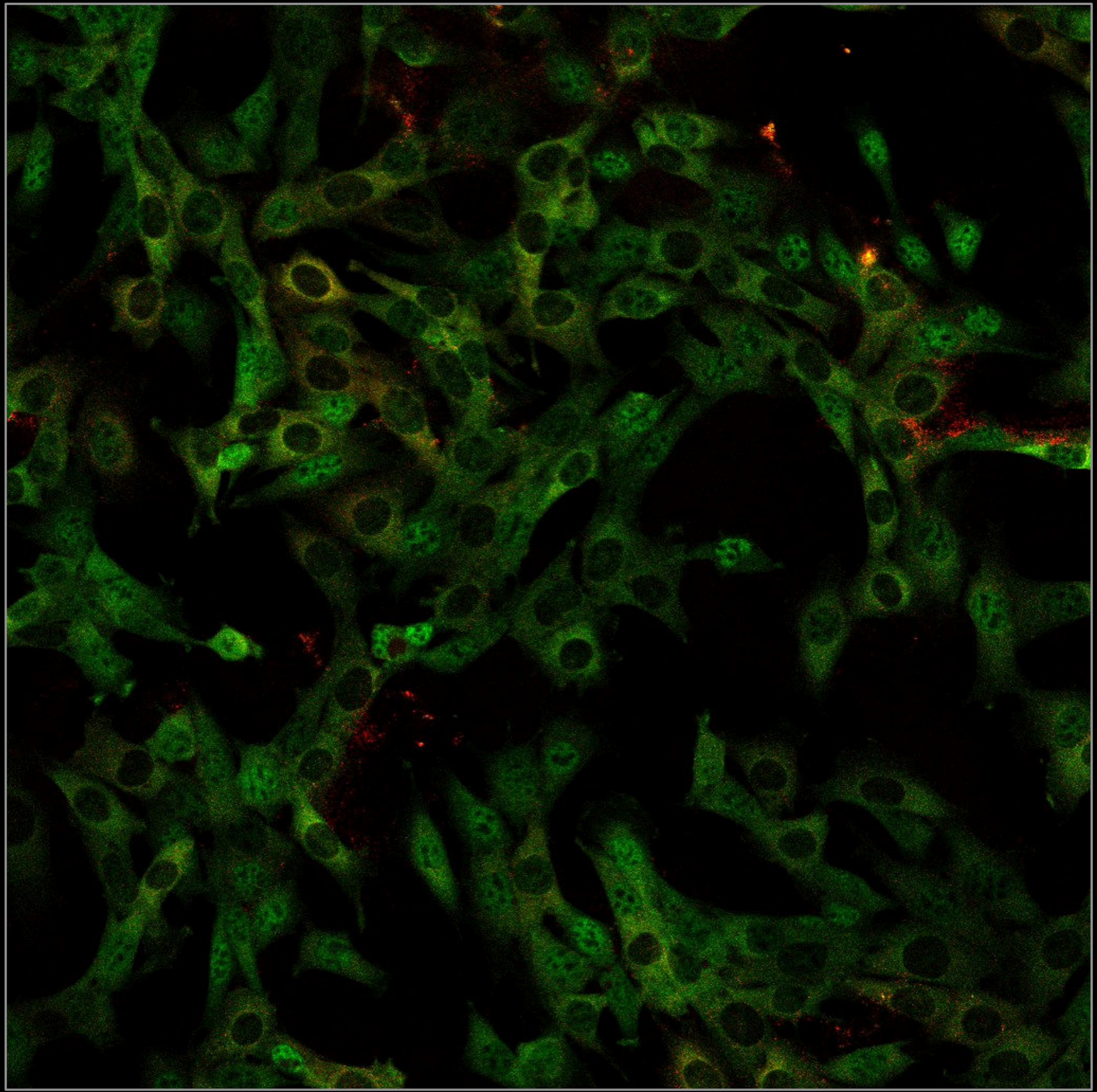

RelA / IκBα

Supplementary Data S3.

MEF + TNF **0.10** ng/ml

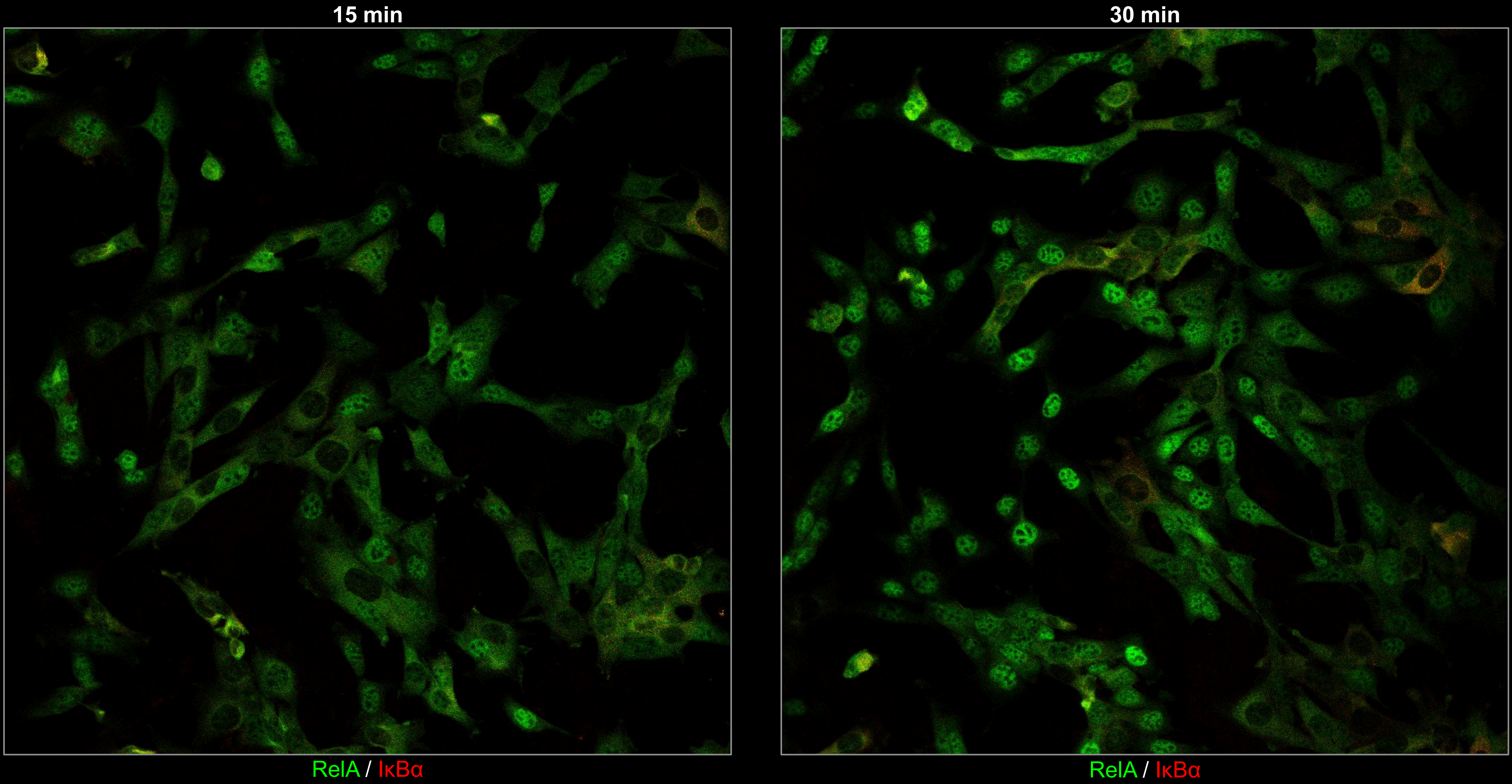

Supplementary Data S3.

MEF + TNF **0.30** ng/ml

15 min **30 min**

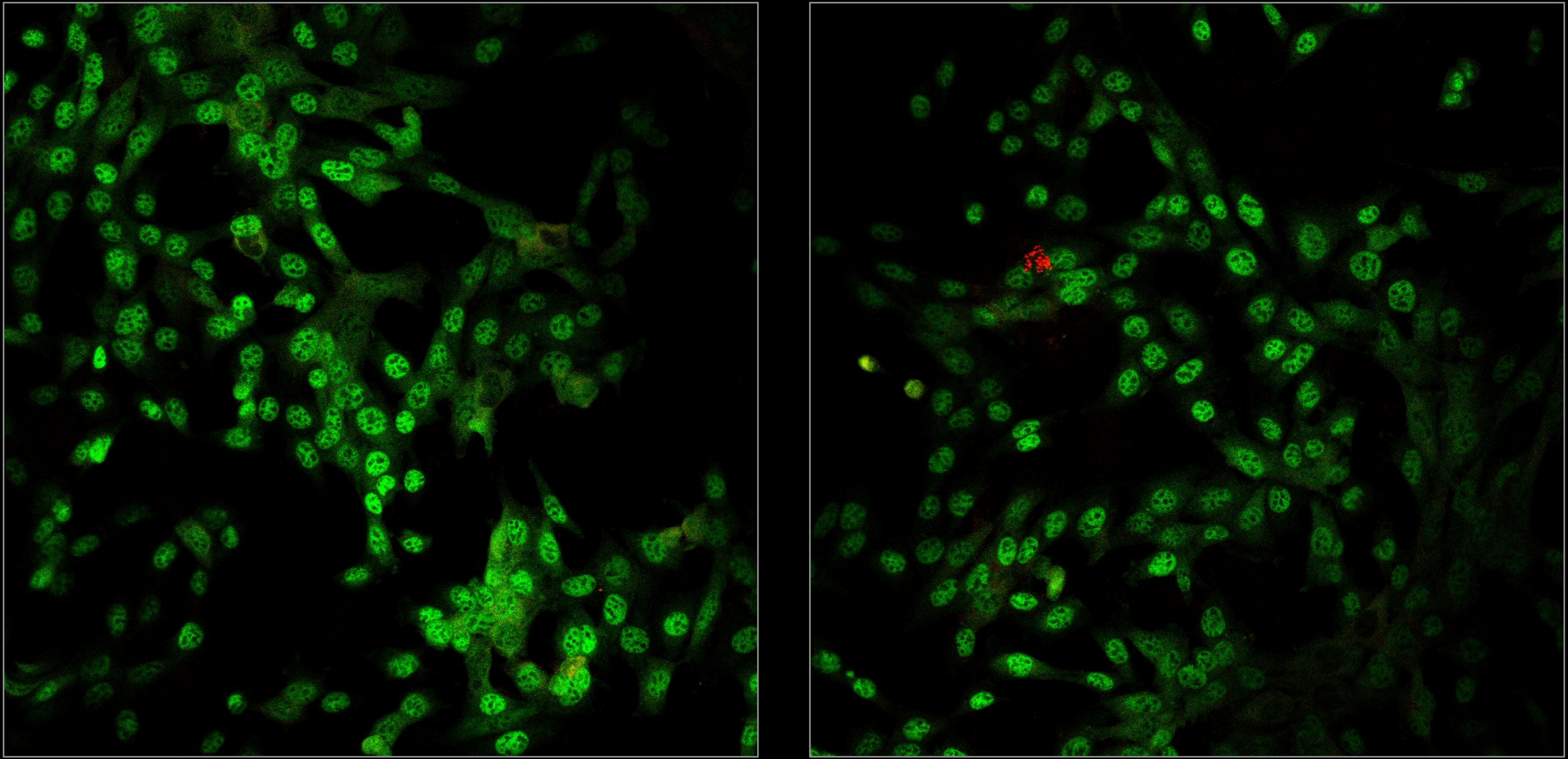

RelA / IκBα RelA / IκBα

Supplementary Data S3.

MEF + TNF **1.00** ng/ml

15 min

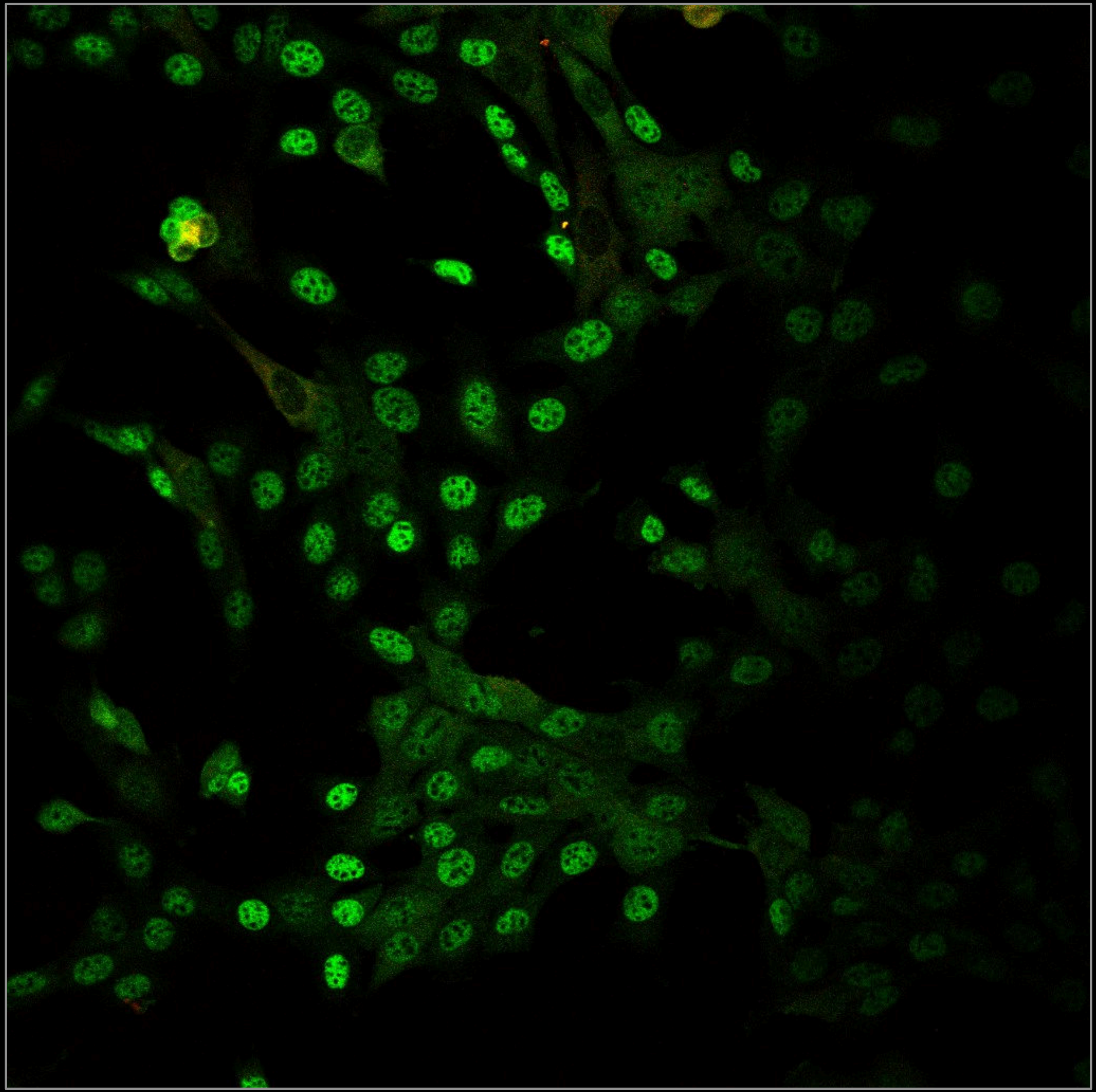

RelA / IκBα

30 min

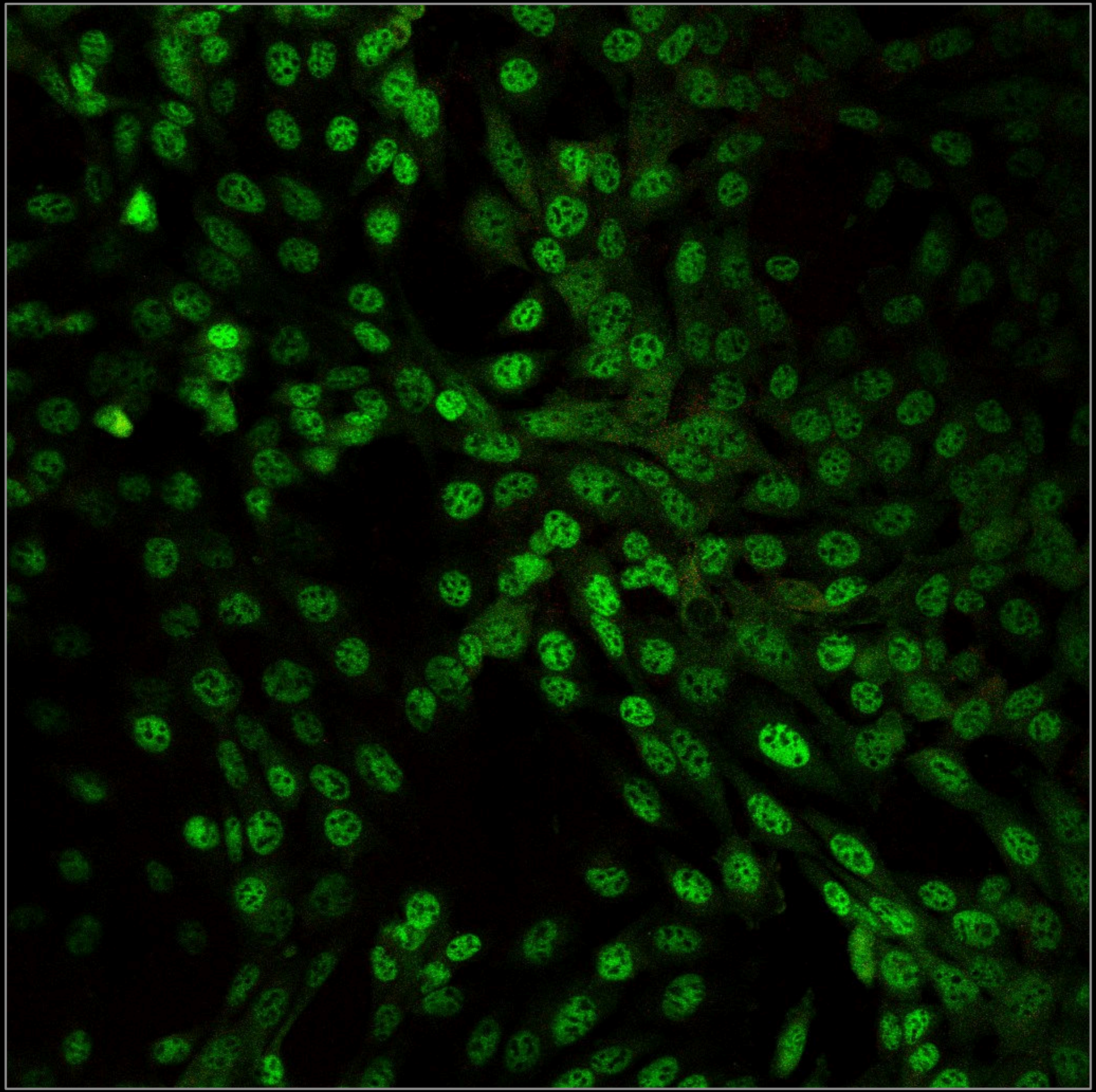

RelA / IκBα

Supplementary Data S3.

MEF + TNF **3.00** ng/ml

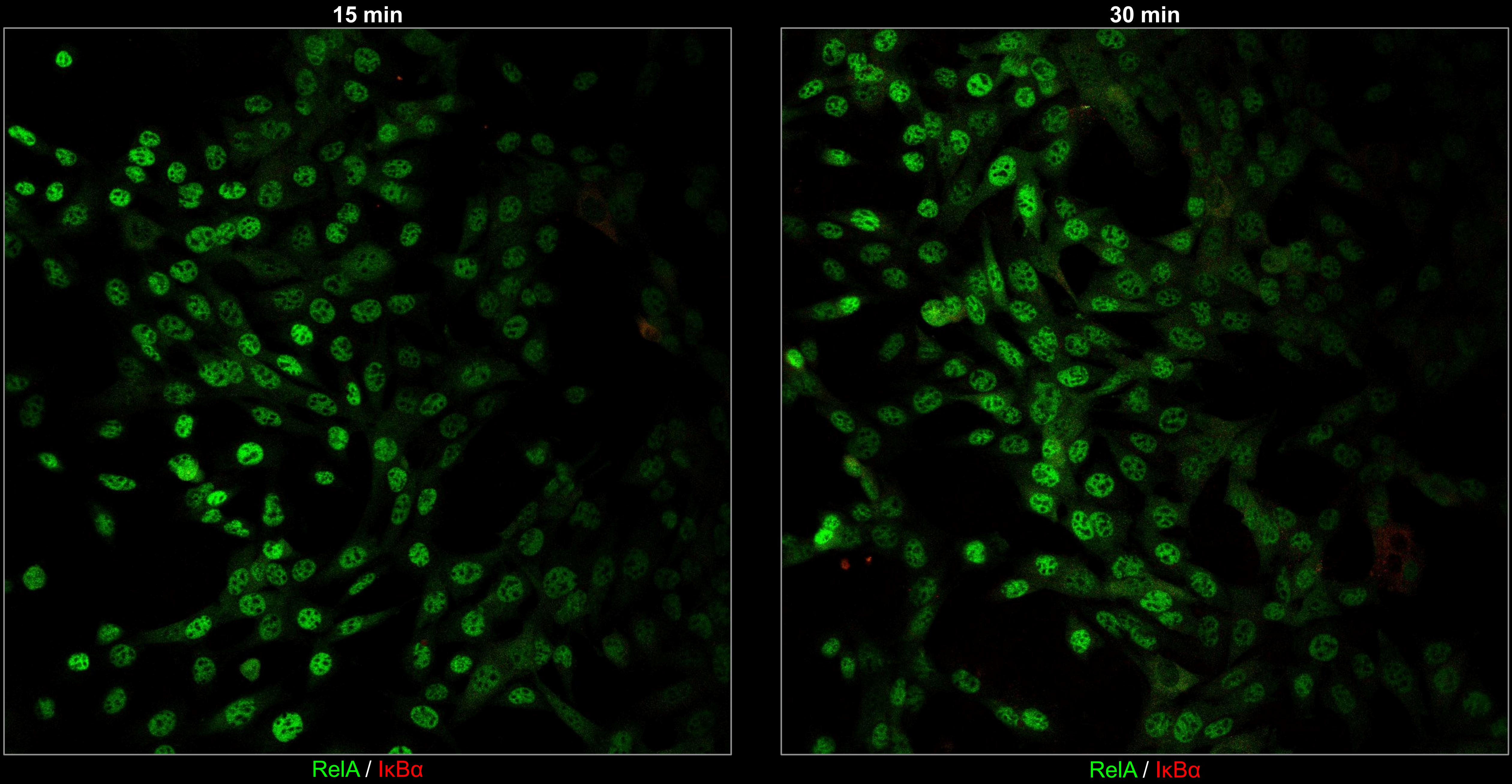

Supplementary Data S3.

MEF + TNF **10.00** ng/ml

15 min

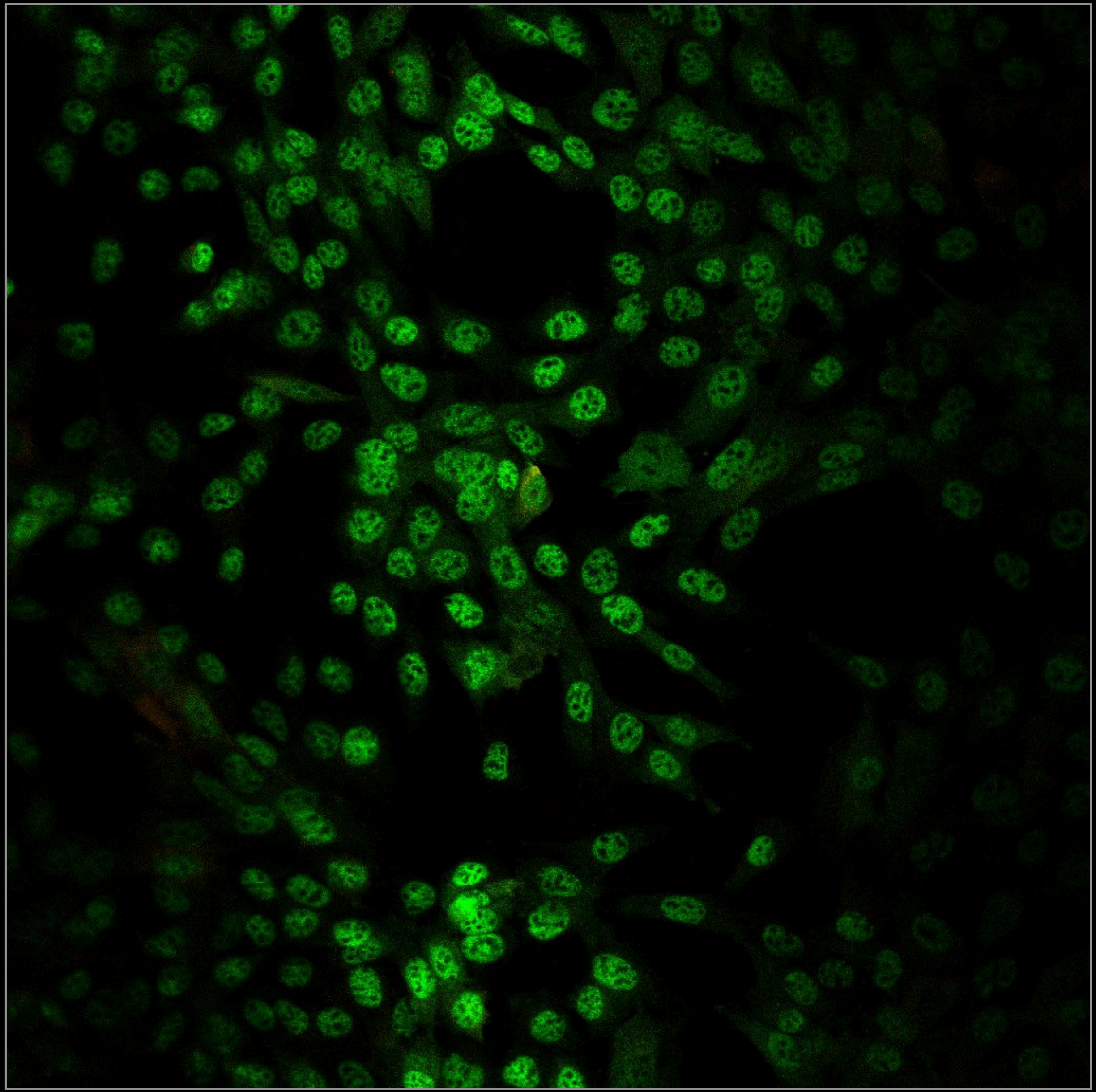

RelA / IκBα

30 min

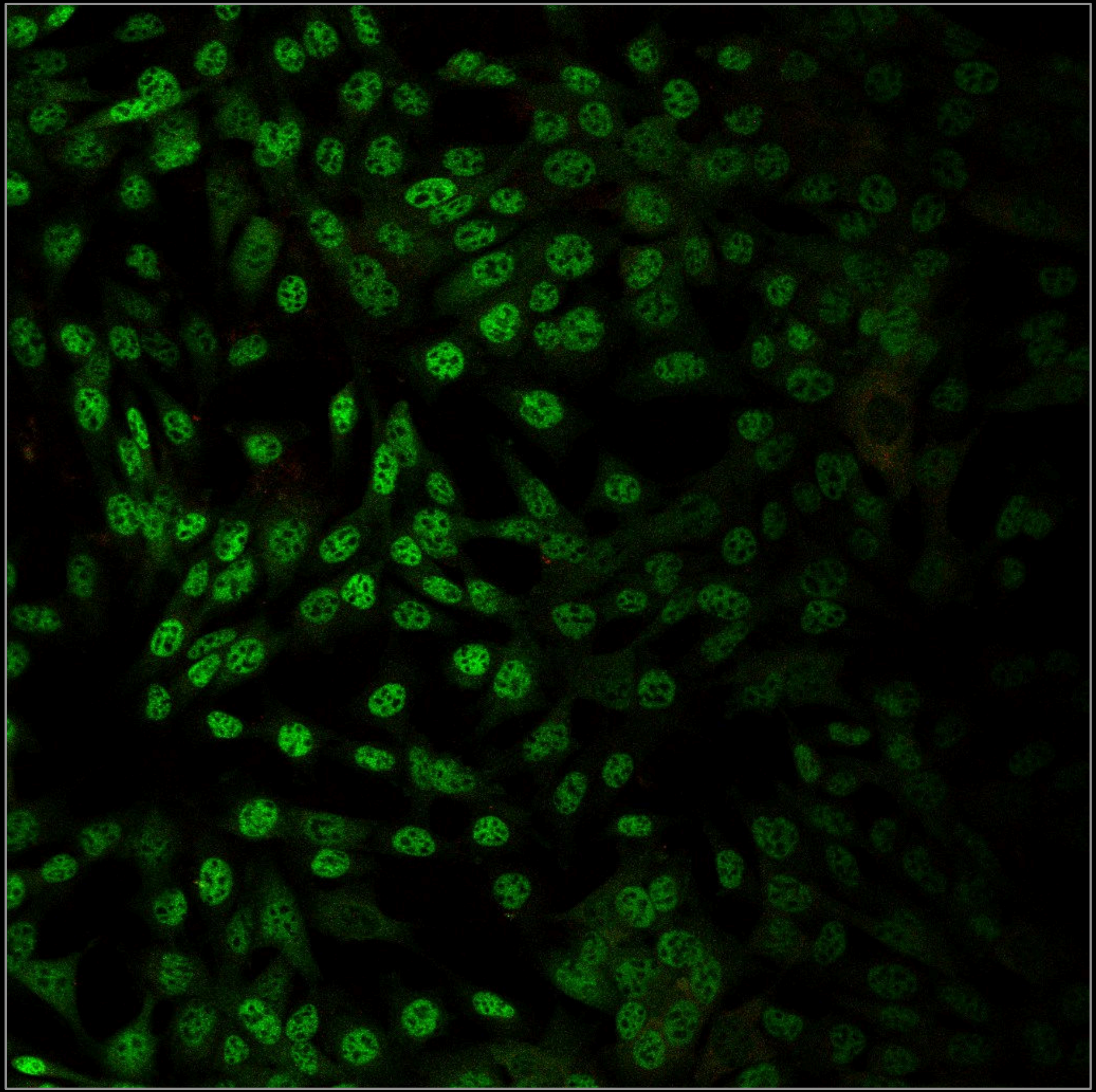

RelA / IκBα

Supplementary Data S4.

Kolmogorov–Smirnov distances and mutual information of NF-κB$_{nuc}$ for all pairs of TNF concentrations.

(**a**, **b**) Tables contain Kolmogorov–Smirnov (KS) distances and maximized mutual information (MI) values for TNF stimulation at 15 min and 30 min. Each KS distance is calculated between the distributions of nuclear NF-κB for two TNF concentrations (as indicated in the table), for a given stimulation time. Each MI is calculated for a pair of concentrations, with input concentration probabilities p and $p_1 = 1 - p$ optimized to obtain a highest value (when one distribution is broader than the other, then MI may assume its maximum for unequal input probabilities). KS distances and MI values correspond to EXPERIMENTAL and SIMULATION histograms shown in Fig. 3. Simulations were performed with the correction for cytoplasmic interference (with CI), each for n = 10,000 cells. (**c**) Scatter plot shows KS versus $\sqrt{\text{MI}}$ calculated from experimental data (green) and from model simulations (pink).

a EXPERIMENT

15 min

KS

TNF	0	0.01	0.03	0.1	0.3	1	3 ng/ml
0.01	0.21						
0.03	0.11	0.15					
0.1	0.52	0.38	0.45				
0.3	0.81	0.74	0.80	0.48			
1	0.79	0.70	0.76	0.41	0.09		
3	0.87	0.82	0.86	0.54	0.09	0.16	
10 ng/ml	0.86	0.80	0.84	0.60	0.15	0.22	0.08

MI

TNF	0	0.01	0.03	0.1	0.3	1	3 ng/ml
0.01	0.05						
0.03	0.00	0.04					
0.1	0.25	0.13	0.21				
0.3	0.63	0.51	0.61	0.20			
1	0.63	0.49	0.58	0.17	0.01		
3	0.74	0.63	0.71	0.31	0.02	0.03	
10 ng/ml	0.72	0.62	0.69	0.31	0.00	0.02	0.01

30 min

KS

TNF	0	0.01	0.03	0.1	0.3	1	3 ng/ml
0.01	0.30						
0.03	0.46	0.20					
0.1	0.70	0.54	0.40				
0.3	0.80	0.66	0.58	0.25			
1	0.90	0.76	0.68	0.33	0.12		
3	0.84	0.69	0.58	0.20	0.08	0.15	
10 ng/ml	0.87	0.73	0.65	0.31	0.09	0.04	0.13

MI

TNF	0	0.01	0.03	0.1	0.3	1	3 ng/ml
0.01	0.09						
0.03	0.19	0.04					
0.1	0.49	0.24	0.13				
0.3	0.65	0.40	0.32	0.04			
1	0.79	0.56	0.45	0.11	0.01		
3	0.71	0.47	0.34	0.05	0.01	0.02	
10 ng/ml	0.74	0.52	0.40	0.10	0.01	0.01	0.02

b SIMULATION with CI, σ = 0

15 min

KS

TNF	0	0.01	0.03	0.1	0.3	1	3 ng/ml
0.01	0.01						
0.03	0.04	0.03					
0.1	0.15	0.14	0.11				
0.3	0.45	0.44	0.42	0.31			
1	0.81	0.80	0.79	0.71	0.47		
3	0.89	0.89	0.87	0.81	0.60	0.17	
10 ng/ml	0.90	0.90	0.88	0.82	0.62	0.21	0.04

MI

TNF	0	0.01	0.03	0.1	0.3	1	3 ng/ml
0.01	0.00						
0.03	0.00	0.00					
0.1	0.04	0.03	0.01				
0.3	0.24	0.23	0.20	0.11			
1	0.64	0.63	0.60	0.49	0.20		
3	0.77	0.76	0.73	0.64	0.35	0.03	
10 ng/ml	0.79	0.78	0.75	0.66	0.37	0.05	0.00

30 min

KS

TNF	0	0.01	0.03	0.1	0.3	1	3 ng/ml
0.01	0.06						
0.03	0.16	0.10					
0.1	0.45	0.39	0.30				
0.3	0.77	0.73	0.65	0.38			
1	0.87	0.84	0.77	0.54	0.19		
3	0.88	0.86	0.78	0.55	0.20	0.02	
10 ng/ml	0.88	0.85	0.77	0.54	0.19	0.02	0.02

MI

TNF	0	0.01	0.03	0.1	0.3	1	3 ng/ml
0.01	0.01						
0.03	0.05	0.02					
0.1	0.24	0.18	0.09				
0.3	0.59	0.52	0.40	0.14			
1	0.74	0.68	0.56	0.28	0.03		
3	0.76	0.70	0.58	0.29	0.04	0.00	
10 ng/ml	0.75	0.69	0.57	0.28	0.03	0.00	0.00

c

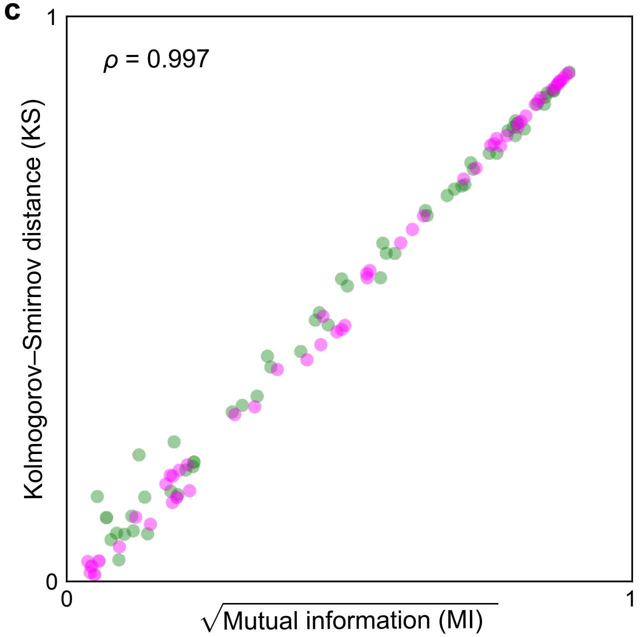

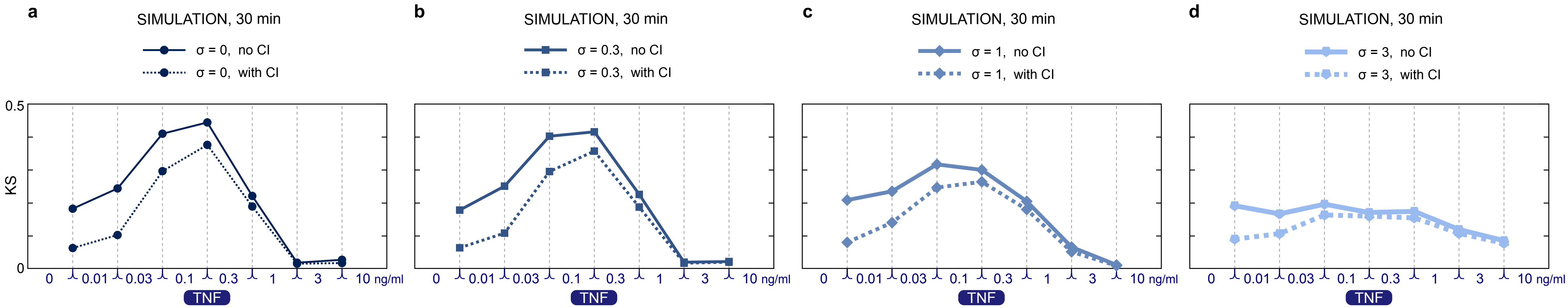

Supplementary Data S5.

The effect of cytoplasmic interference on Kolmogorov–Smirnov distances analyzed based on numerical simulations.

Plots (**a**–**d**) show Kolmogorov–Smirnov (KS) distances between simulated distributions of nuclear NF-κB 30 min since the beginning of stimulation with one of each two consecutive TNF concentrations (as presented in Fig. 4a in the main text). Each plot represents a set of simulations with a different standard deviation of TNFR, σ, and juxtaposes results with and without cytoplasmic interference (labeled with CI and no CI, respectively). See Fig. 1 and Methods in the main text for more details on CI.

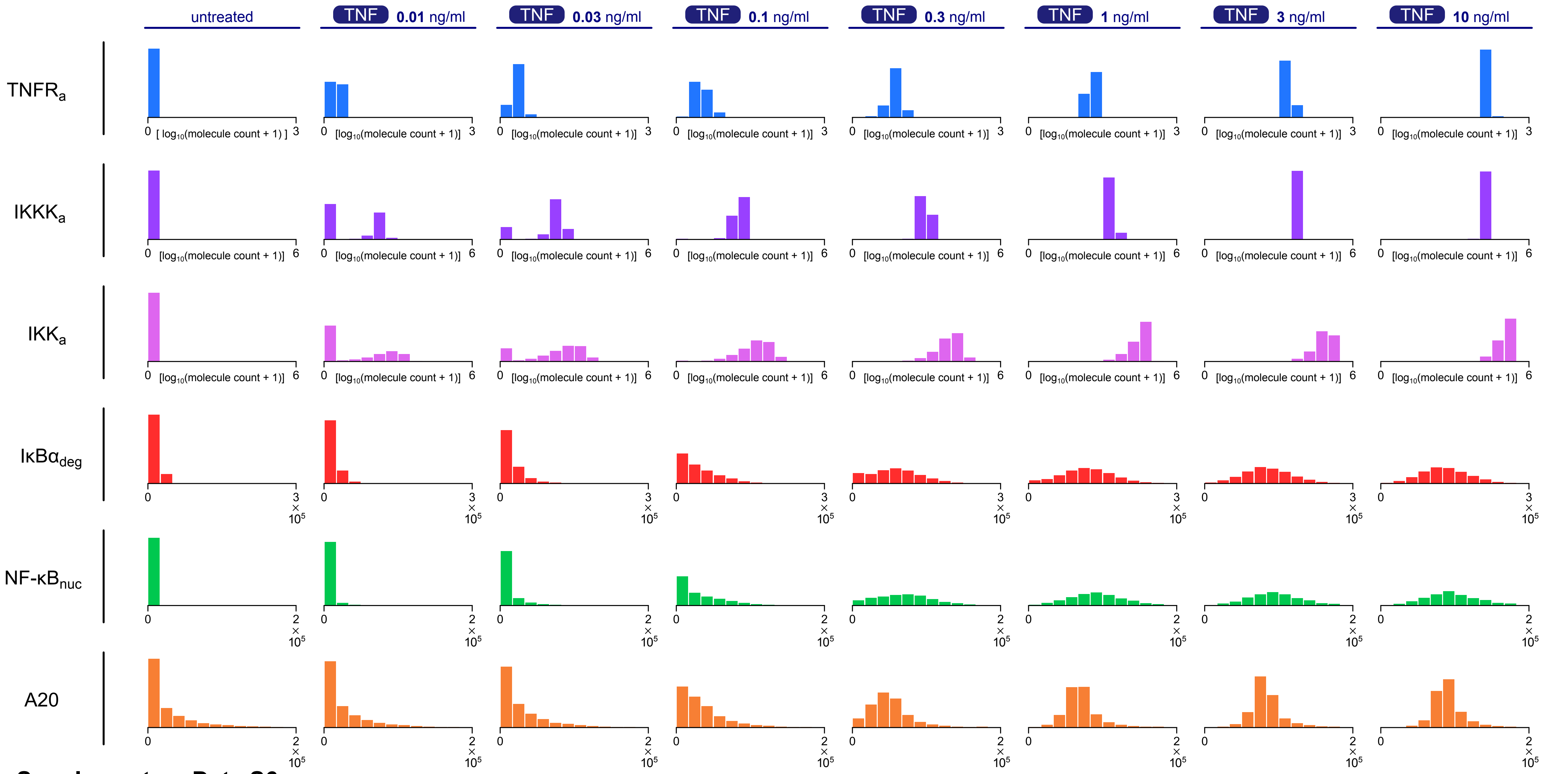

Supplementary Data S6.

Histograms of pathway components after stimulation with eight TNF concentrations. Results were based on n = 10,000 SIMULATIONs with the number of TNFR equal $2{\times}10^3$ (σ = 0). Maximal protein levels from the first 30 min after stimulation were analyzed. Nuclear NF-κB was calculated without correction for cytoplasmic interference (no CI).

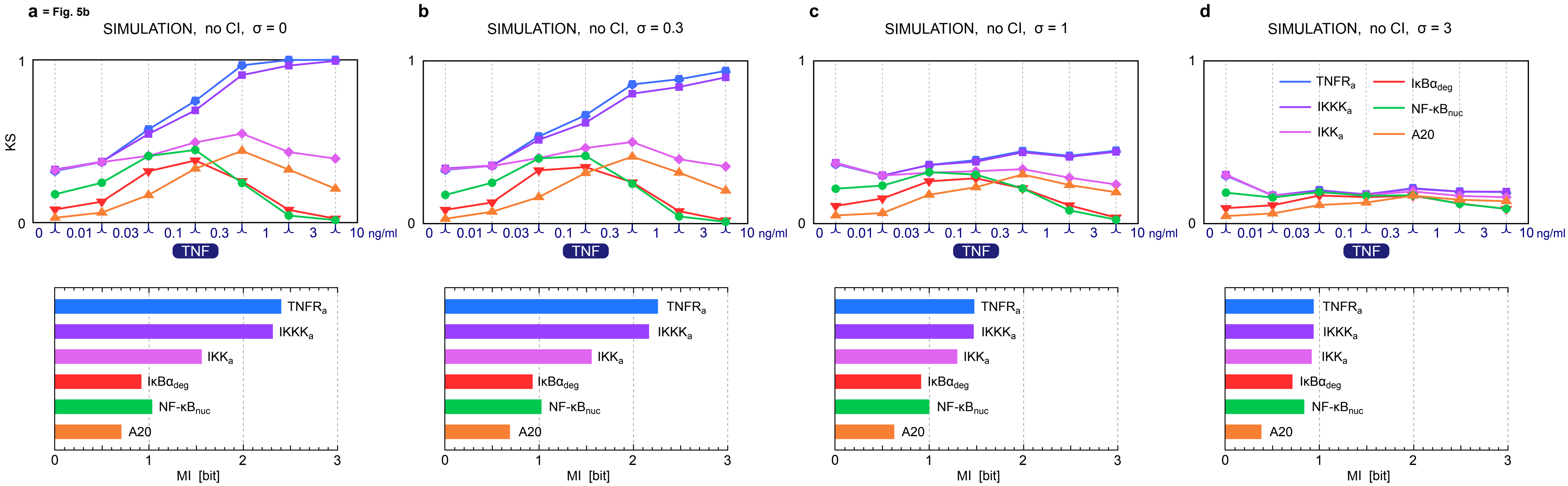

Supplementary Data S7.

The effect of extrinsic noise on Kolmogorov–Smirnov distances and mutual information.

(**a**–**e**) Upper line-plots show Kolmogorov–Smirnov (KS) distances between simulated distributions of pathway components for each two consecutive TNF concentrations; lower bar-plots show mutual information (MI) on the level of each pathway component.

The results are based on maximal levels of pathway components over 0–30 min after TNF stimulation, for n = 10,000 simulations with (**a**) σ = 0, (**b**) σ = 0.3, (**c**) σ = 1, (**d**) σ = 3 and (**e**) for intrinsic noise only, all with no correction for cytoplasmic interference (no CI).

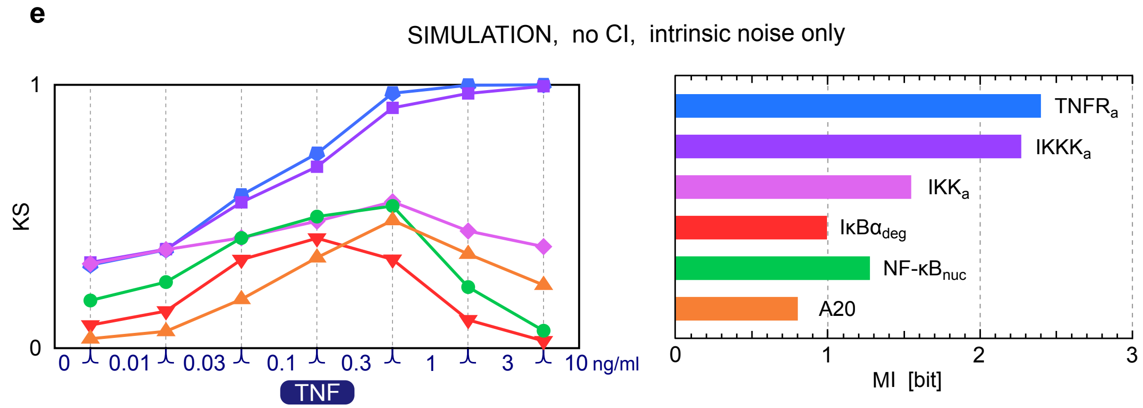

Supplementary Data S8.

Full frames of immunofluorescent confocal images showing MEFs stimulated with LPS or costimulated with CHX and LPS.

Immunofluorescent confocal images used to obtain data shown in Fig 6. MEFs were stimulated with 1 μg/ml LPS in the absence or presence of 5 μg/ml CHX. In the CHX+LPS costimulation experiment, incubation with 5 μg/ml CHX started 30 min before TNF stimulation. Cells were then fixed and stained with antibodies for RelA (a subunit of NF-κB; green) at 0 (untreated), 30 and 120 min after LPS stimulation.

Supplementary Data S8.

0 min (control)

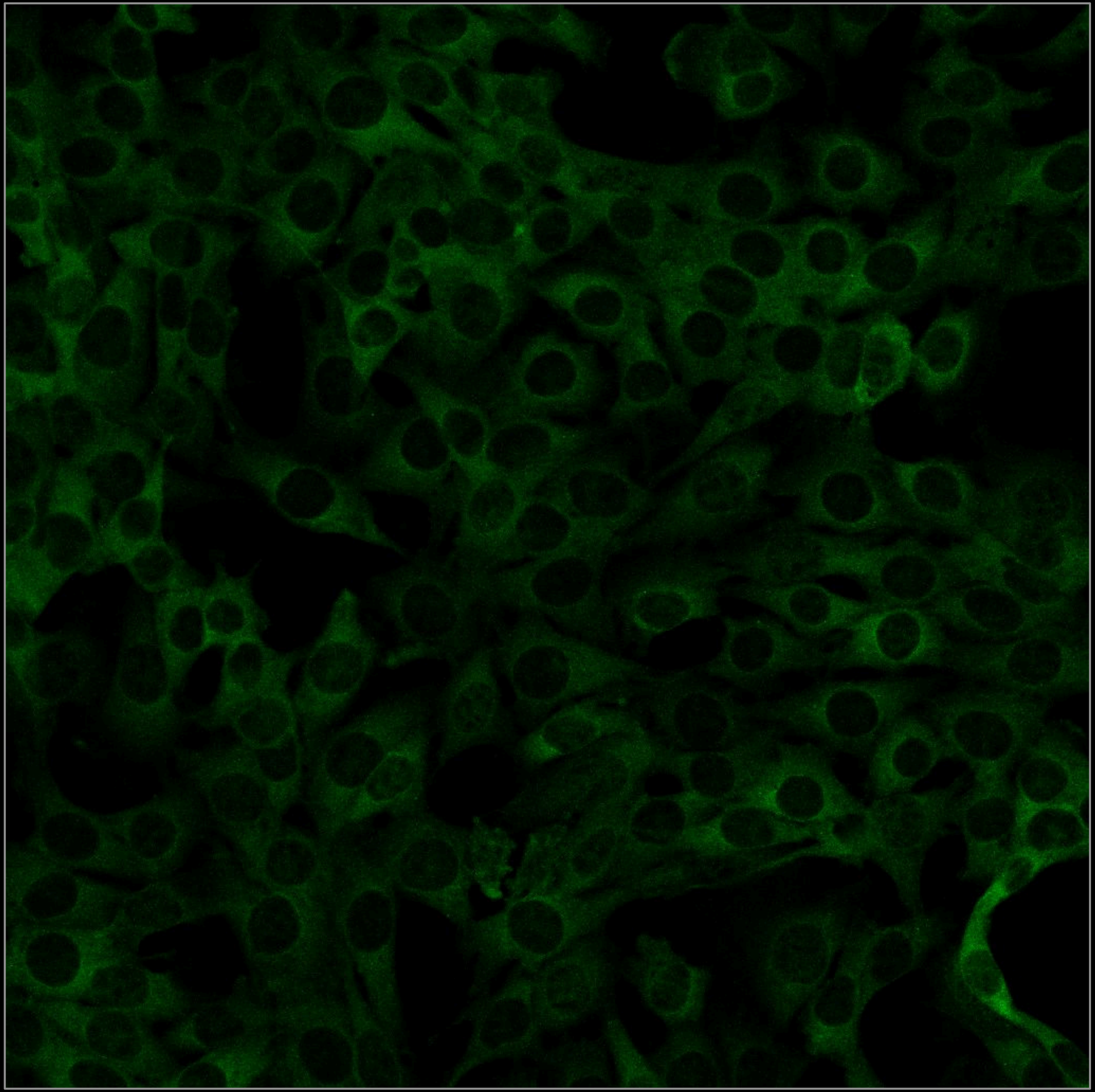

RelA

Supplementary Data S8.

30 min CHX 5 μg/ml

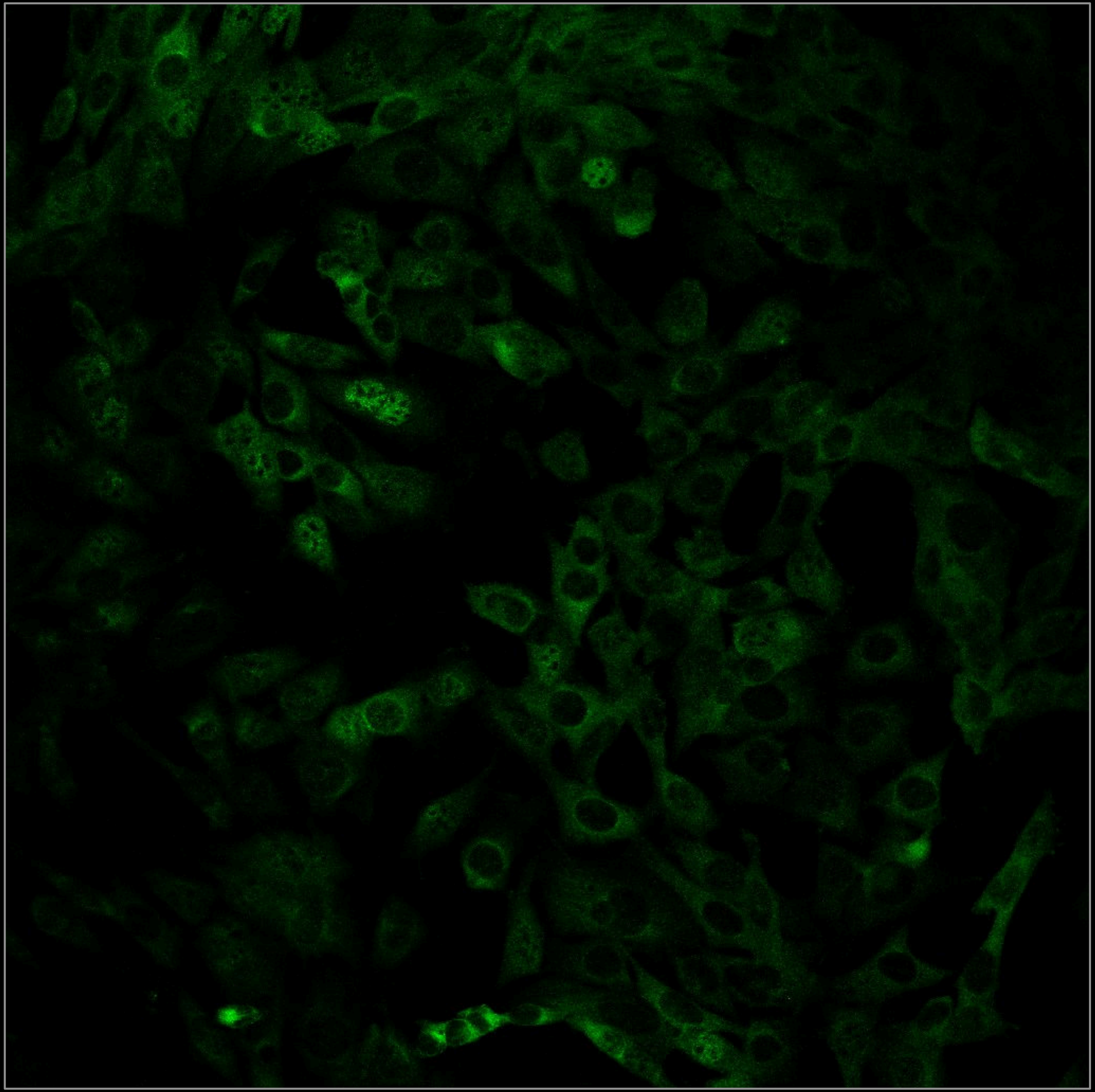

Supplementary Data S8.

30 min LPS 1 μg/ml

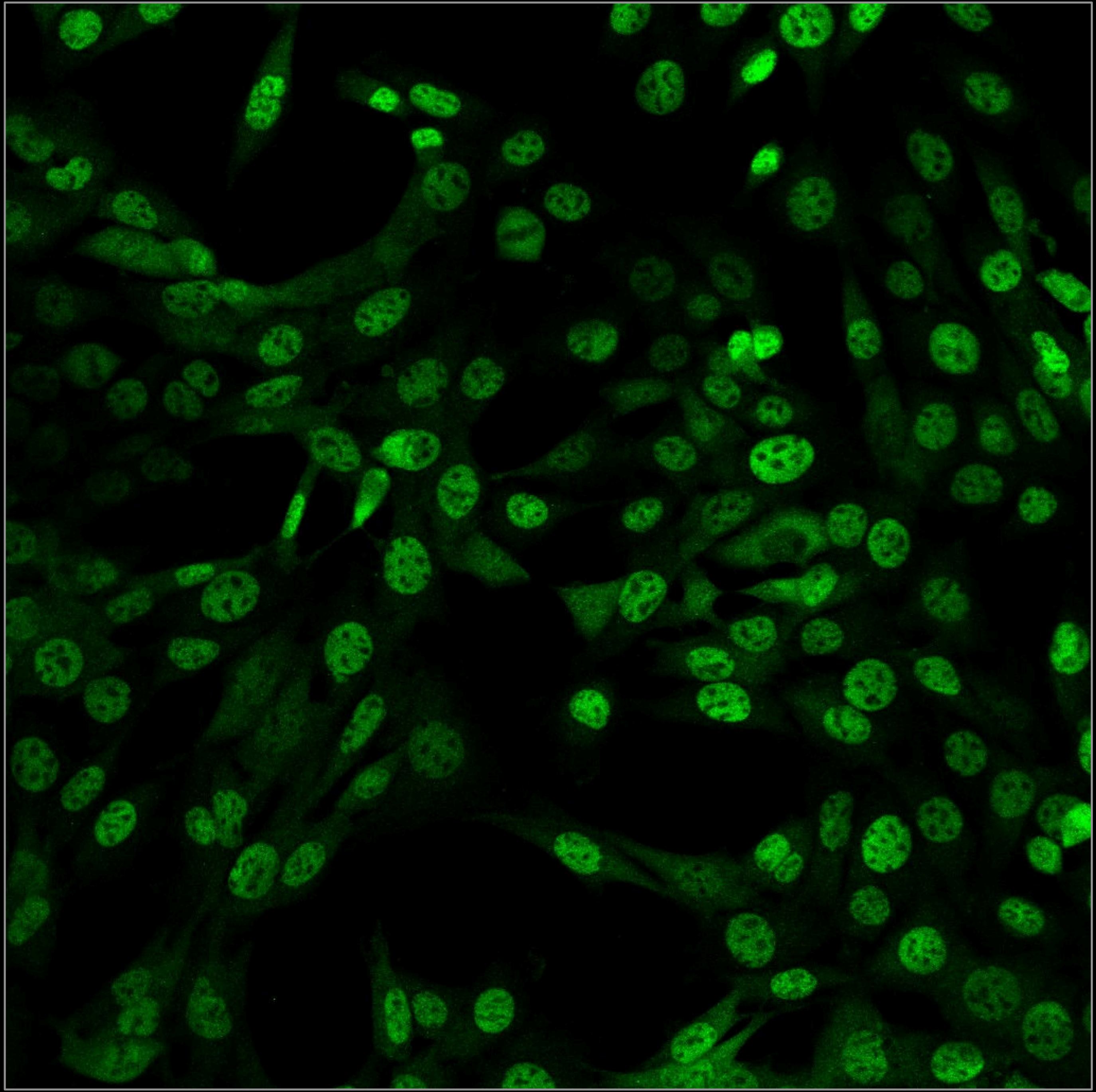

Supplementary Data S8.

CHX+LPS costimulation:

5 μg/ml CHX added
30 min before LPS,

30 min LPS 1 μg/ml

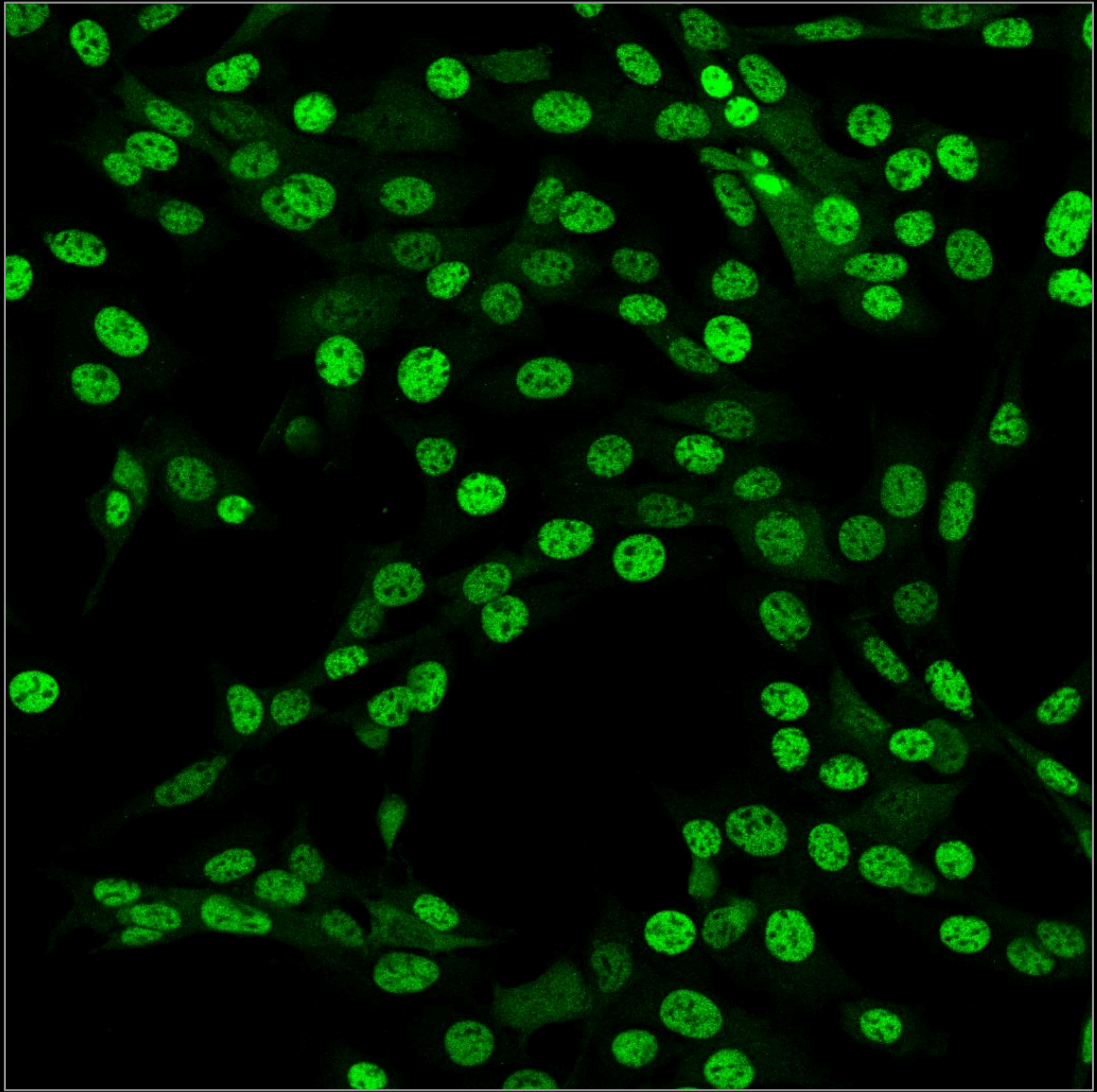

RelA

Supplementary Data S8.

120 min CHX 5 μg/ml

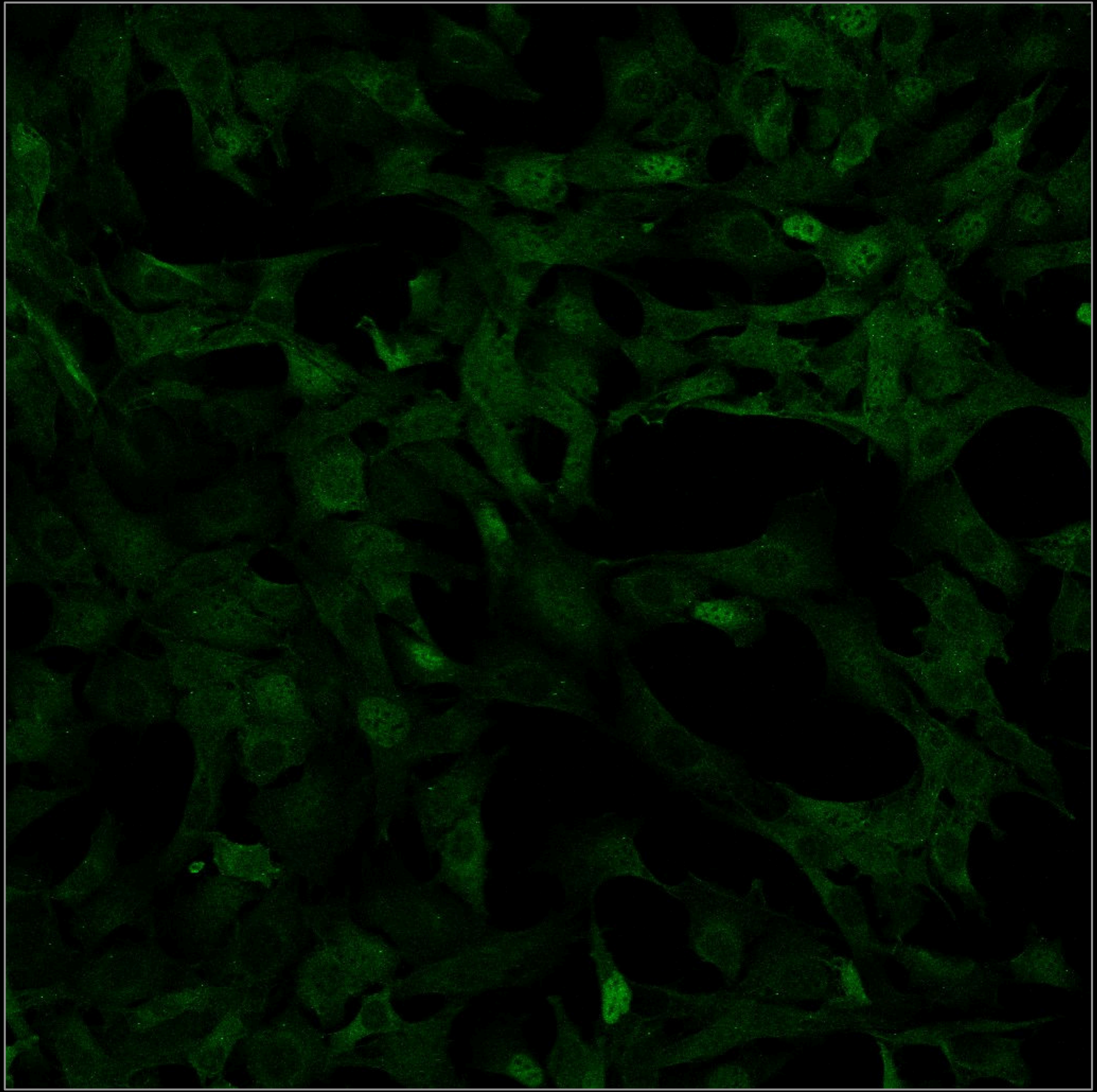

RelA

Supplementary Data S8.

120 min LPS 1 μg/ml

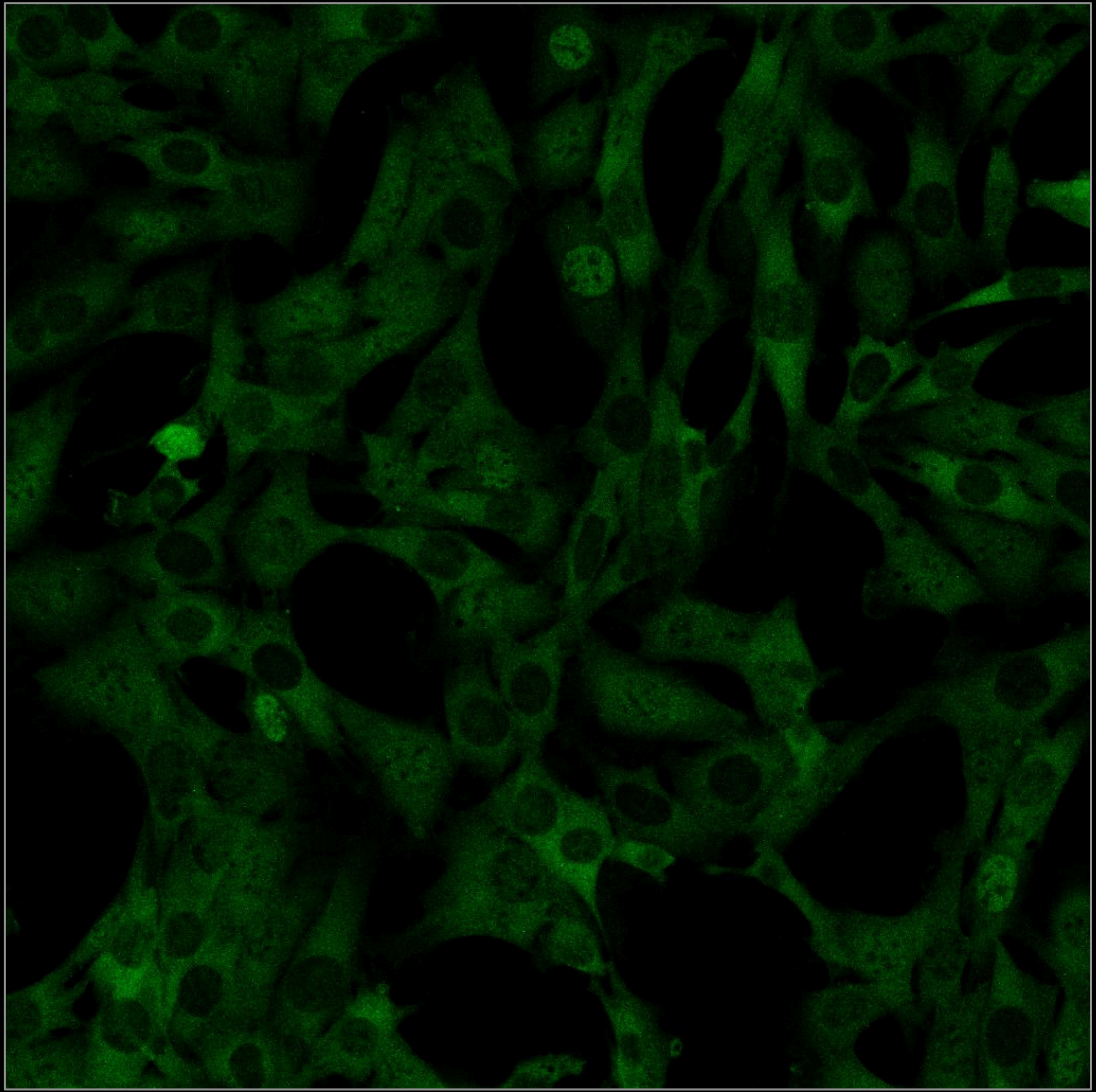

RelA

Supplementary Data S8.

CHX+LPS costimulation:

5 μg/ml CHX added
30 min before LPS,

120 min LPS 1 μg/ml

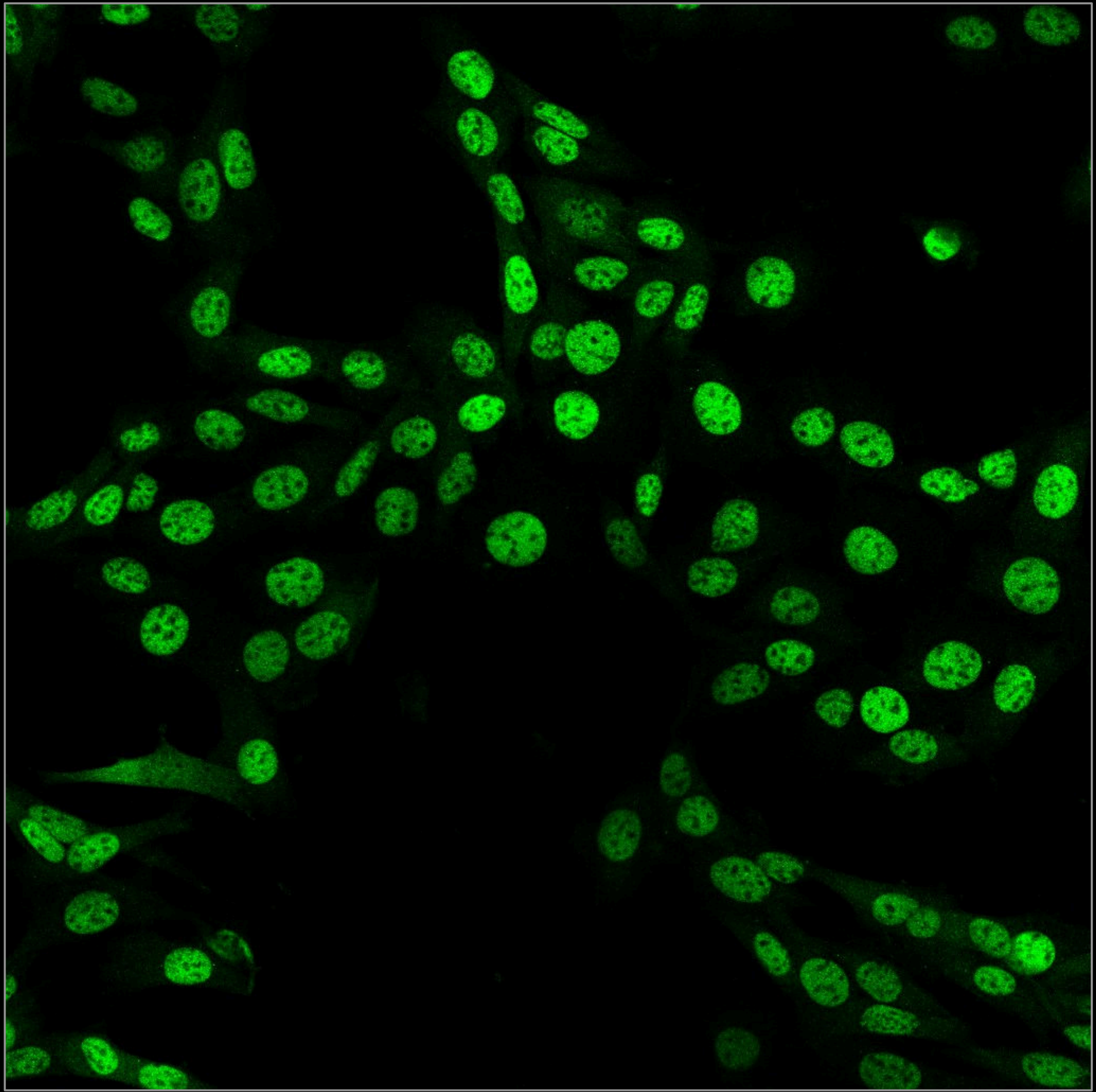

These two supplementary materials are provided as separate files:

Supplementary Dataset S1.

Model implementation in BioNetGen language.

When executed in BioNetGen, it is used to perform a simulation with conditions identical to those in the right subpanels of Fig. 5a (3 ng/ml TNF, $\sigma = 0$) for the pool of translocatable NF-κB = 10^5.

Supplementary Dataset S2.

Python implementation of the Kraskov algorithm for estimating mutual information.

When executed, the code performs a validation on a set of samples from 8 adjacent, partially overlapping Gaussian distributions for three values of *k* for *k* nearest-neighbor search. Example output is provided as a TXT file. Reference results of maximal mutual information computed in Wolfram Mathematica are provided in the form of a Mathematica notebook (NB) file and its export to PDF.
