# Supplementary material for: Information processing in the NF-κB pathway
Source: Sci Rep. 2017 Nov 21;7:15926. doi: 10.1038/s41598-017-16166-y (PMC5698458; doi:10.1038/s41598-017-16166-y)
Supplement: Supplementary file 3 — Dataset 2 [file 41598_2017_16166_MOESM3_ESM.zip › SupplementaryDatasetS2/MutInfoGauss8x.pdf]

Maximal mutual information for overlapping Gaussians

Definitions

Mutual information

```
Ent[P_, weights_List, i_] :=
    weights[[i]] P[i] Log2[P[i]/Sum [weights[[j]] P[j], {j, Length[weights]}]];
MutInfo[P_, weights_, Lbound_, Ubound_] :=
  Sum [NIntegrate[Ent[P, weights, i], {x, Lbound, Ubound}], {i, 1, Length[weights]}]
```

Gaussian probability densities

```
μ[i_] = 1.5 i;
σ[i_] = 1 + i/4;
Gauss[i_] := PDF[ NormalDistribution[μ[i], σ[i]] ] [x];
```

The considered case of 8 Gaussians

```
n = 8;
Lbound = μ[1] - 5 σ[1];
Ubound = μ[n] + 5 σ[n];
Plot[Evaluate[Table[Gauss[i], {i, 1, n}]], {x, Lbound, Ubound}, PlotRange → Full]
```

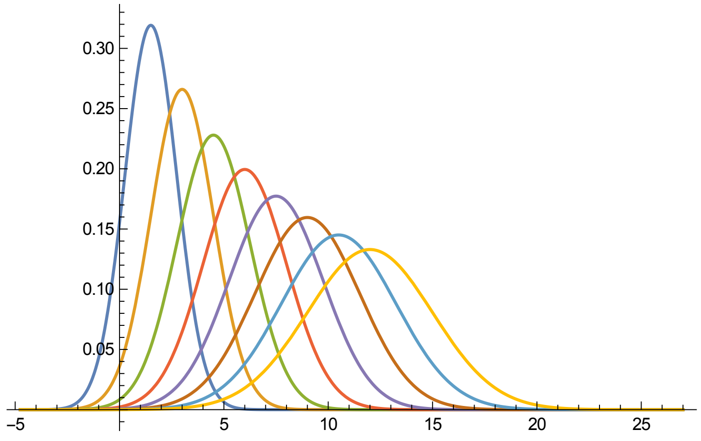

Evaluation of mutual information

```
MutInfo[weights_? (VectorQ[#, NumericQ ] &)] := MutInfo[Gauss, weights, Lbound, Ubound]
```

1) Mutual information for equal weights

```
equalW = Table[1/n, {n}];
MIequalW = MutInfo@equalW;
Print["MI = ", MIequalW, " at weights ", equalW, "."]
```

MI = 0.935408 at weights $\left\{\frac{1}{8}, \frac{1}{8}, \frac{1}{8}, \frac{1}{8}, \frac{1}{8}, \frac{1}{8}, \frac{1}{8}, \frac{1}{8}\right\}$.

2) Mutual information for “antipodal” weights

```
antipodalW = {1/2}~Join~Table[0, {n-2}]~Join~{1/2};
MIantipodalW = MutInfo@antipodalW // Quiet;
Print["MI = ", MIantipodalW, " at weights = ", antipodalW, "."]
```

MI = 0.971205 at weights = $\left\{\frac{1}{2}, 0, 0, 0, 0, 0, 0, \frac{1}{2}\right\}$.

3) ***Maximal mutual information*** *from constrained optimization of weights*

```
weights = Table[w[i], {i, n}];
maxMIwithOptW =
    FindMaximum [{MutInfo[weights], (* This takes 2-3 minutes . *)
        Table[0 ≤ weights[[i]] ≤ 1, {i, n}], Total[weights] == 1}, weights];
MImax = First@maxMIwithOptW ;
MImaxW = Table[Round[w[i], 10^-5] /. First@Rest@maxMIwithOptW , {i, n}] // N;
Print["Max MI = ", MImax , " at weights = ", MImaxW , "."]
```

Max MI = 1.14425 at weights = {0.38722, 0., 0.0464, 0.21112, 0., 0., 0., 0.35526}.
